# Tri-partite complex for axonal transport drug delivery achieves pharmacological effect

**DOI:** 10.1186/1471-2202-11-8

**Published:** 2010-01-20

**Authors:** Aaron G Filler, Garth T Whiteside, Mark Bacon, Martyn Frederickson, Franklyn A Howe, Miri D Rabinowitz, Alan J Sokoloff, Terrence W Deacon, Chris Abell, Raj Munglani, John R Griffiths, B Anthony Bell, Andrew ML Lever

**Affiliations:** 1 Institute for Nerve Medicine, 2716 Ocean Park Blvd., Suite 3082, Santa Monica, CA, 90405, USA; 2 Department of Cell and Molecular Biology, St. George's Hospital Medical School, University of London, Cranmer Terrace, London, SW17 ORE, UK; 3 Department of Neurosurgery, Atkinson Morley's Hospital, St. George's, University of London, 31 Copse Hill Road, London SW20 ONE, UK; 4 Division of Neurosurgery, UCLA School of Medicine, Center for Health Sciences, 10833 Le Conte Ave, Los Angeles, CA, 90095, USA; 5 SynGenix LTD, Babraham Hall, Babraham Research Campus, Cambridge, CB22 3AT, UK; 6 Molecular Synthetics LTD, Babraham Hall, Babraham Research Campus, Cambridge, CB22 3AT, UK; 7 Wyeth Research, Neuroscience Discovery Research, CN 8000 Princeton, NJ, 08543, USA; 8 Spinal Research, Station Road, Bramley, Guildford, Surrey, GU5 0AZ, UK; 9 Astex Therapeutics, 436 Cambridge Science Park, Milton Road, Cambridge, CB4 0QA, UK; 10 Carmell Therapeutics Corporation, 10 South Tower, 320 E. North Ave., Pittsburgh, PA 15212, USA; 11 Department of Physiology, Emory University School of Medicine, 615 Michael Street, Atlanta, GA 30322, USA; 12 Department of Organismic & Evolutionary Biology, Museum of Comparative Zoology, Harvard University, 26 Oxford Street, Cambridge, MA, 02138, USA; 13 Department of Anthropology, Human Evolutionary Biology, Peabody Museum, Harvard University, 11 Divinity Ave., Cambridge, MA, 02138, USA; 14 Department of Anthropology, University of California at Berkeley, 232 Kroeber Hall, Berkeley, CA 94720, USA; 15 Neuroscience Unit, St. George's University of London, Cranmer Terrace, London, SW17 ORE, UK; 16 Molecular Imaging, Cancer Research UK Cambridge Research Institute, Robinson Way, Cambridge, CB2 ORE, UK; 17 Department of Anesthesia, Addenbrooke's Hospital, University of Cambridge, Hills Road, Cambridge, CB2 2QQ, UK; 18 Department of Chemistry, University Chemical Laboratory, University of Cambridge, Lensfield Road, Cambridge, CB2 1EW, UK; 19 Department of Internal Medicine, Addenbrooke's Hospital, University of Cambridge, Hills Road, Cambridge, CB2 2QQ, UK

## Abstract

**Background:**

Targeted delivery of pharmaceutical agents into selected populations of CNS (Central Nervous System) neurons is an extremely compelling goal. Currently, systemic methods are generally used for delivery of pain medications, anti-virals for treatment of dermatomal infections, anti-spasmodics, and neuroprotectants. Systemic side effects or undesirable effects on parts of the CNS that are not involved in the pathology limit efficacy and limit clinical utility for many classes of pharmaceuticals. Axonal transport from the periphery offers a possible selective route, but there has been little progress towards design of agents that can accomplish targeted delivery via this intraneural route. To achieve this goal, we developed a tripartite molecular construction concept involving an axonal transport facilitator molecule, a polymer linker, and a large number of drug molecules conjugated to the linker, then sought to evaluate its neurobiology and pharmacological behavior.

**Results:**

We developed chemical synthesis methodologies for assembling these tripartite complexes using a variety of axonal transport facilitators including nerve growth factor, wheat germ agglutinin, and synthetic facilitators derived from phage display work. Loading of up to 100 drug molecules per complex was achieved. Conjugation methods were used that allowed the drugs to be released in active form inside the cell body after transport. Intramuscular and intradermal injection proved effective for introducing pharmacologically effective doses into selected populations of CNS neurons. Pharmacological efficacy with gabapentin in a paw withdrawal latency model revealed a ten fold increase in half life and a 300 fold decrease in necessary dose relative to systemic administration for gabapentin when the drug was delivered by axonal transport using the tripartite vehicle.

**Conclusion:**

Specific targeting of selected subpopulations of CNS neurons for drug delivery by axonal transport holds great promise. The data shown here provide a basic framework for the intraneural pharmacology of this tripartite complex. The pharmacologically efficacious drug delivery demonstrated here verify the fundamental feasibility of using axonal transport for targeted drug delivery.

## Background

The direct distribution of medications to specific target tissues in the central nervous system (CNS) is an attractive objective for the treatment of problems such as local pain or dermatomal viral infection. When systemic drug delivery is used to treat a symptom affecting a small segment of the nervous system (such as pain in a single dermatome) conventional drug delivery methods can be highly inefficient because they place most of the dose in non-neuronal tissues throughout the body and in areas of the CNS that do not need treatment. Dose limitation that hinders efficacy often arises from the concentration of the drug in non-target tissues. For many compounds, this situation is aggravated because access to tissues of the central nervous system is also compromised by the relative impermeability of the blood brain barrier.

The nervous system itself provides an alternative to the vascular system or the cerebro-spinal fluid (CSF) - the intra-axonal route. However, this has proven difficult to exploit. Methods using direct injection into nerves [1], introduction of modified neurotropic viruses [2], or delivery of neurotoxins such as tetanus toxin or ricin [3,4] have been explored but each pose barriers to routine clinical use. Direct injection may damage nerves, neurotropic viruses expose the recipient to direct damage or the risk of recombination with wild type virus, and toxins for nerve destruction have limited clinical roles. Phage display has been used to generate synthetic peptides to promote axonal transport [5], but it has not been clear how to exploit this.

Retrograde axonal transport of exogenous molecules from the periphery to CNS neuronal cell bodies is long established [6] and is one of the methodological bases for mapping neuroanatomical pathways [7-9]. The underlying physiological basis of this process and its biochemistry are increasingly well understood [10-19]. However, although axonal transport for drug delivery has been proposed previously [20-24], little or nothing is known about the major features of this biological system from the point of view of approaching it pharmacologically and there has been no agent demonstrated capable of accomplishing a pharmacological effect via this route.

Pharmacological entrainment of axonal transport is acknowledged as a highly desirable objective since it would allow for targeted antiviral, antineuropathic or regenerative treatments to segmentally selected ganglion or CNS cells. We sought to clarify the pharmacology of intraneural drug delivery and to design agents capable of accomplishing therapeutic tasks by clinically applicable axonal transport methods employing intramuscular injection and subsequent uptake by intact nerve endings.

**Figure 1:**
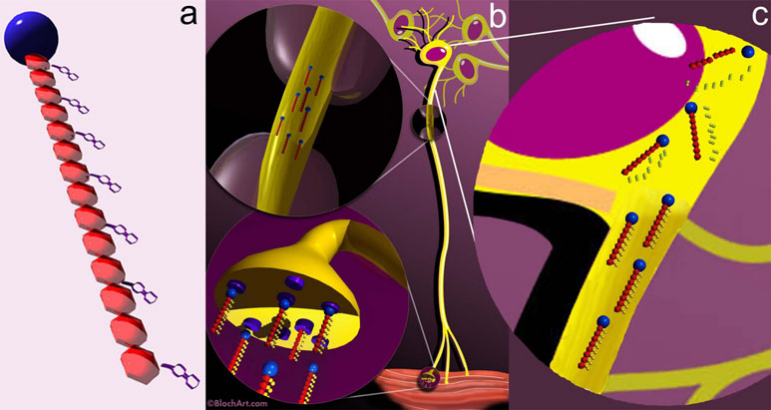
**Tripartite delivery vehicle**. (**a**) The drug delivery vehicles include a targeting element - the axonal transport facilitator or ATF (blue sphere), and a polymer such as dextran (red repeating units) that carry multiple drug molecules (purple). (**b**) They are designed to be injected in muscle or skin and then travel via an "intraneural" route to deliver the drug molecules to the cell body. (**c**) Late during transport and on arrival in the cell body, lytic processes release the active drug molecule by breakdown of the linker components.

We designed a novel tripartite molecular complex to provide a flexible architecture capable of achieving this. We now report on this class of pharmacological agents for selective targeted access and transport through the nervous system and demonstrate that it is capable of achieving therapeutic efficacy. The system provides a versatile platform for many different therapies with the same targeting requirements. The agents used are various forms of a tripartite complex composed of a first moiety acting as an "axonal transport facilitator" (ATF), an "amplifying polymer" (AP) second moiety acting to achieve amplification of the fundamental event of synaptic endocytosis by carrying along multiple drug molecules with each saturable uptake event, and a third moiety composed of multiple copies of the therapeutic molecule, reversibly linked to the polymer (Figure 1). This novel molecular architecture allowed us to independently explore and optimize general features and constraints affecting the three major aspects of the problem - A) chemical synthesis, B) optimization of uptake and transport, and C) intraneuronal drug efficacy after transport.

We used a comprehensive array of biological, physical and chemical assays *in vitro *and *in vivo *to characterize and optimize the system. The studies are numbered [1] to [23] corresponding to the summarized results table (table 1).

**Table 1:** Axonal transport experiments

	Study	Agent used	Model	Evaluation method	Result	Figure	Impact
**A Synthesis, function and stability**							

							

**I Chemical synthesis**							

							

	1	Dextran tripartites	Chemical	NMR, PLC, Mass Spec	30% loading	2	

		a. CM dex standard					

		b. CM dex-amino upgraded					

		c. CM dex-lysine extended					

		d. WGA-CM dex					

		e. WGA-CM dex-valacyclovir					

		f. CM dex-gabapentin					

		g. WGA-CM dex-gabapentin				1	Validate chemical basis of uptake amplification

							

	2	Ferrite particle tripartites	chemical	MRI	useful effect on T2 at obtainable conc.		

		a. dex coated particles precip					

		b. periodate conjugation					

		c. affinity purification					Validate assembly of nanoparticulate carrier

		d. DNA adhesion					

		e. relaxivity assessment		test tube cast gels	solenoid coil	7	Validate imageability in MRI

							

**II Intracellular release and drug activation**							

							

	3	valacyclovir	BHK-Herpes	plaque count	control		Establish cellular capability of uptake and release

		dex-val	BHK-Herpes	plaque count	steric decrease in uptake		

		WGA-dex-val	BHK-Herpes	plaque count, assay	improved delivery		

							

**B Interaction with Axon Terminals and Intra-axonal environment**							

							

**III Effects of polymer, linker and drug**							

							

	4	Dextran 10K	symp gang	fluor mic	rapid uptake		Evaluate effects of tripartite size on uptake

		Dextran 70K	symp gang	fluor mic	slower uptake		

							

	5	Dextran-FITC neut	symp gang	fluor mic	extensive uptake	3	Evaluate effects of tripartite charge on uptake

		Dextran-FITC pos	symp gang	fluor mic	extensive uptake		

		Dextran-FITC neg	symp gang	fluor mic	limited uptake		

							

	6	Acylation	symp gang	fluor mic	no uptake		Evaluate effects of hydrophobicity on uptake

							

**IV Effects of Axonal Transport Facilitator**							

							

	7	WGA-FITC	symp gang, Campenot	fluor mic	WGA and NGF well transported		Comparability of physiologic and non-phys ATF in vitro

		NGF-TR	symp gang, Campenot	fluor mic	WGA and NGF well transported	4,17,18	

							

	8	Phage display	Campenot	pfu's	identification of synthetic ATF		Phage display for generation of synthetic ATFs

							

	9	Transport inhibitor	symp gang, Campenot	fluor mic	blocked by inhibitors of transport		Confirmation of role of microtubule based transport

							

**V Effects of Intra-axonal Processing**							

							

	10	Anti-gabapentin Ab	gastroc/ant tib - rat	excise, Ab stain	delivery of gabapentin antigenicity to DRG		Confirm delivery of gabapentin antigenicity

							

	11	WGA-HRP	axial - M. fascicularis	HRP-TMB backlit	small IM inj to enzyme in cells in primates	5	Confirm that enzymatic function survives transport

							

	12	WGA-dex-mag	gastroc - rabbit	EM	transport in axoplasm	6	Verify axonal transport compartment

							

	13	59-Fe WGA-dex-mag	gastroc/ant tib-rabbit	autoradiography	gross sections		Confirm intraneural transport of particle tripartite

							

	14	WGA-dex-mag	gastroc/ant tib-rabbit	MRI vs gel chamber	non-lysis of particle	8,19	Confirmation of transport of intact magnetite

							

	15	WGA-dex-mag	forearm-rabbit	4.7T high res MRI	demonstration of nerve image	9	Confirmation of fast transport of intact magnetite

							

**C Targeting and pharmacological efficacy**							

							

**VI Clinical target access**							

							

	16	WGA-FITC	hindlimb - rat	excise, section, fl mic	ventral horn & intermediolateral	10	Assess access to relevant motor & autonomic targets

		WGA-dex-FITC	hindlimb - rat	excise, section, fl mic	ventral horn & intermediolateral		

		Dex-FITC	hindlimb - rat	excise, section, fl mic	limited transport		

							

	17	WGA-FITC	hindlimb - rat	excise, section, fl mic	DREZ lamina I and II	11	Confirm access to dorsal root entry zone

		WGA-FITC	hindlimb - rat	excise, section, fl mic	DRG axon dendrite		

							

	18	WGA-FITC	hindlimb - rat	fluor mic, cell count	good filling in DRG	12,13	Assess properties of access to dorsal root ganglia

		WGA-TRITC	foot pad-rat	fluor mic, cell count	good filling in DRG		

							

	19	WGA-FITC	hindlimb - rat	fluor mic, peripherin	nociceptors reached	14	Verify access to nociceptors

		dextran-FITC	hindlimb - rat	fluor mic, peripherin	no transport		

							

**VII Distinctive pattern of distribution relative to trans-vascular**							

							

	20	125-I WGA	forelimb/hindlimb - rat	dissect, count	saturable, time consistent, selective	15	Characterize expected whole body distrib

		125-I NGF	hindlimb - rat	dissect, count	differences in distribution due to ATF		Evaluate effects of ATF on distribution

		59-Fe WGA-dex-mag	forelimb - rat	dissect, count	large particles comparable		Evaluate effects of large agent size on distribution

							

	21	131-I WGA	gastroc/ant. tib-rabbit	gamma camera image	voxel size causes averaging and inj site bright		Examine local injection area effects

							

	22	14C-gbp-dex	hindlimb - rat	dissect, count	minimal		Validate delivery of therapeutic dose

		14C-gbp-dex-WGA	hindlimb - rat	dissect, count	transported in high conc.		

							

**VIII Unique pharmacologic effects not obtainable by trans-vascular**							

							

	23	gbp-dex-WGA	foot pad/hindlimb - rat	withdrawal latency	prolonged decrease in hyperalgesia	16	Validate pharmacological efficacy

		gbp-dex	foot pad/hindlimb - rat	withdrawal latency	minimal	16	

		gbp	foot pad/hindlimb - rat	withdrawal latency	minimal	16	

		gbp-dex-WGA	cervical fat pad - rat	withdrawal latency	minimal	16	

A) We studied the synthesis and stability of the tripartite and its components addressing the following questions: [*study 1*] Can tripartite molecules be constructed chemically to preserve efficient adsorptive endocytosis when loaded with large numbers of conjugated drug molecules? [*study 2*] What are the upper size limits for the tripartite complex? [*study 3*] Can pharmacologic activity be preserved for small molecules released from the tripartite complex under intracellular conditions?

B) We analyzed interactions with axon terminals and the intra-axonal environment that would affect design of the tripartite by investigating the following questions: [*studies 4, 5 & 6*] - How do polymer size, molecular charge, and hydrophilicity affect the efficiency of uptake into neurons? [*studies 7, 8 & 9*] - What is the relative efficiency of physiologic ATFs (axonal transport facilitators) when compared to non-physiologic ATFs for uptake and transport? Can purely synthetic ATF's be produced by phage display techniques that will be more effective than physiologic ATFs? Will use of colchicine - an inhibitor of axonal transport serve to verify interpretations? [*studies 10,11,12,13,14 & 15*] Can a small intramuscular or intradermal injection cause significant delivery into a selected set of neurons via intact uninjured axon terminals in normal tissues? Can this clinically usable method of drug administration achieve high intra-neuronal drug concentrations when compared to the "cut neuron" methods used to introduce axonal transport tracer molecules in some anatomical studies? During axonal transport, if the pharmacologic agents are sealed inside vesicles, will the drug or carrier be damaged or destroyed by lysosomal activity while traveling long distances in the intracellular space of the axons?

C) We investigated targeting and pharmacological efficacy: [*studies 16,17,18 & 19*] - Which classes of clinically useful sub-targets in the nervous system can be reached by clinically convenient administration techniques? [*studies 20 & 21*] - What are the unique features of the whole body pharmacologic distribution of intraneuronal agents and how do size of the molecular complex and selection of ATF affect the distribution? [*studies 22 & 23*] Can pharmacologically efficacious doses of drugs be delivered and are they functional when delivered to the interior of a cell rather than to its exterior surface?

Using a tripartite construct designed according to the results of the investigations outlined above, we were able to administer a small intramuscular injection with 1/300^th ^of the usual oral dose of gabapentin (half life 8 hours) and achieve a degree of neuropathic pain suppression that could not be obtained with tolerable amounts of oral therapy while extending the half life of the drug by more than an order of magnitude.

## Results

Results are summarized in table 1

### Chemical synthesis and stability (aspect A)

#### Chemical entities (project I)

##### Effective synthesis with loading sufficient for amplification [study 1]

**Figure 2:**
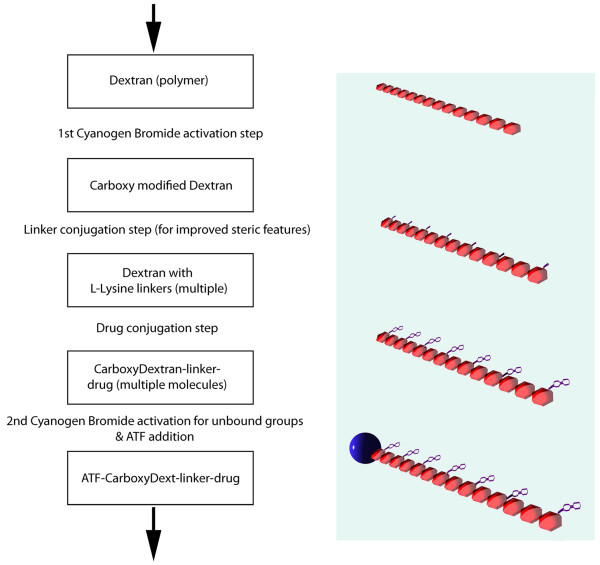
**Outline of conjugation and synthesis**. A polymer such as dextran is first activated with cyanogen bromide to generate a carboxy modified dextran that can be conjugated to linker molecules attached at each monomer. The linkers are then conjugated with drug molecules. A second cyanogen bromide activation step is then carried out and followed by conjugation with an axonal transport facilitator molecule (ATF). An affinity purification step is then used to obtain drug loaded polymer complexes with ATF attached.

The synthesis strategy (Figure 2) achieved attachment of drug to at least 30% of dextran monomers for both gabapentin and valacyclovir. The 70,000 MW dextran has approximately 430 dextrose sub-units so each Wheat germ agglutinin (WGA) molecule endocytosed in these experiments delivered around 100 molecules of drug.

##### Particle based carriers can be delivered intraneuronally [study 2]

EDTA washing of dextran coated magnetite particulates resulted in hydroxide free ferrites that were non-reactive and preserved their superparamagnetic properties during axonal transport. After conjugation of an axonal transport facilitator to the dextran coat, they were effectively endocytosed and transported intact despite their (10-15 nm) size (size measured by electron microscopy).

#### Intracellular release and drug activation (project II)

##### The stability of a drug is maintained through chemical linkage and biological release [study 3]

In the BHK (Baby Hamster Kidney cell line) viral plaque reduction experiments, free drug in the culture medium resulted in plaque reduction by 96% at a dose of 5 micrograms/well, but a dose of 50 micrograms/well of the drug bound to dextran was required to achieve an equivalent effect. However we observed nearly 100% plaque reduction using a dose of 5 microgram/well of tripartite WGA-dextran-drug conjugate. This demonstrates that the WGA efficiently promoted endocytosis of the tripartite agents while dextran-drug alone was not readily taken up by cells.

On chemical grounds, the carboxyl links binding drug molecules to dextran should be hydrolyzed after endocytosis. For valacyclovir (pharmacologically inactive), the product of the hydrolysis is free and active acyclovir. Therefore, the antiviral effect of the tripartite confirms release of acyclovir from the tripartite carrier after endocytosis.

### Interactions with axon terminus and axonal processing (aspect B)

#### Effects of polymer, linker and drug (project III)

##### Effects of polymer size on tripartite uptake into nerves [study 4]

For the two dextran sizes tested, the uptake was faster for the smaller 10K molecular weight dextran than for the larger 70K molecular weight dextran. However, uptake of the 70K molecule was more effective from the point of view of drug delivery. This is because each 70K dextran delivered more attached drug molecules (100 drug molecules per 70K dextran polymer chain as opposed to 14 drug molecules delivered on each 10K dextran polymer chain).

With 10K dextran, there is extensive uptake within ten minutes after application and washing. Similar levels of uptake are seen with 70K dextran after 60 minutes. However the differential efficacy of uptake was not large enough to outweigh the benefits of greater amplification of uptake events achieved by using the higher capacity larger molecules for drug delivery. Since molecular loading efficiency of the polymers was equivalent for various sized dextrans, the increased drug delivery with increased molecular size was linear. However, the losses in uptake efficiency with larger molecules was not severe enough to make them less effective overall. The consequence is that although smaller dextran molecules were endocytosed more avidly and rapidly than 70K based vehicles, more drug molecules were still delivered by the 70K based vehicles if a full hour was allowed to elapse after introduction of the test agent. Larger molecule sizes were not tested.

##### Effects of charge on uptake and transport [study 5]

**Figure 3:**
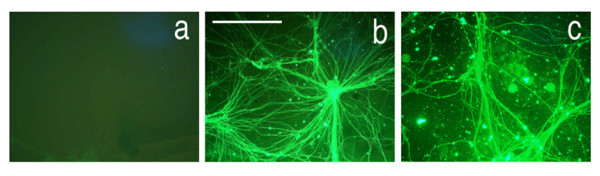
**Effects of molecular charge on neuronal uptake and transport**. (**a**) lack of uptake of WGA-dextran-FITC after carboxyl derivatization resulting in negative charge.(**b**) good uptake with neutral charge from 50:50 carboxyl and amine derivatization. (**c**) good uptake with positive charge from amine derivatization. Scale bar is 120 μm.

The overall charge of the complex had a large impact on efficiency of uptake (Figure 3). There was virtually no uptake into cultured neurons when the overall charge of the complex was negative, and effective uptake occurred when charge was neutral, or when overall charge was positive. This finding parallels the natural movement of positively charged ions into the negatively charged internal milieu of the cell during depolarization. Similar charge findings have been reported for liposomal drug delivery systems [25].

##### Effects of side group mediated hydrophobicity on uptake and transport [study 6]

Acylation of FITC (fluorescein isothiocyanate)-labeled, WGA-conjugated dextran to make the molecules progressively hydrophobic led to complete failure of uptake and transport. We assessed various degrees of acylation and found that this effect occurred even at very low ratios of acylation.

#### Effects of axonal transport facilitator (project IV)

##### Efficacy of physiologic, non-physiologic and synthetic ATFs [studies 7,8,9]

**Figure 4:**
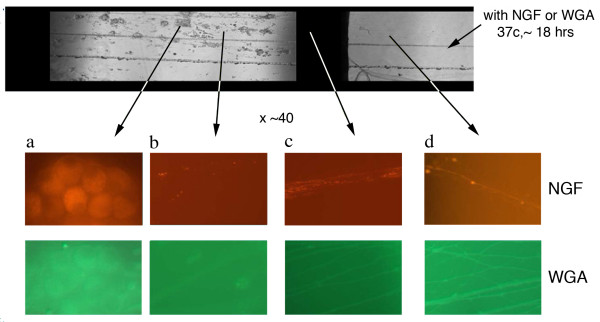
**Axonal Transport of NGF and WGA in Campenot Chambers**. Top panel shows a transmission image of the central and side compartments of a Campenot chamber. WGA-FITC or NGF-Texas Red were added to the side compartment and left overnight. At higher magnification, the "red series" of panels are fluorescent images obtained from the arrowed areas of the chamber following NGF-TR administration. Similarly, the "green" series of panels shows fluorescent images taken of the chambers following WGA-FITC administration. The left most panels in both series show uptake of fluorescence in cell bodies derived from the axonal transport of these labeled ATFs.

The Campenot chamber studies demonstrated similar efficacy for Nerve growth factor (NGF) and WGA for promoting uptake of the tripartite carrying FITC (Figure 4). The phage display experiments demonstrated that novel purely synthetic ATFs could be discovered and then produced *in vitro *which not only had equivalent efficiency for neuronal uptake, but which could also be more efficiently sub-targeted to different types of neuronal populations (e.g. general sensory, pain, motor, autonomic).

When colchicine - an inhibitor of axonal transport - was added to Campenot chambers, it blocked intra-axonal movement of molecular complexes whose ATF was derived from purely synthetic phage display techniques. This confirms that the drug transport effect produced by the synthetic phage display products achieved delivery by axonal transport.

#### Effects of intra-axonal processing (project V)

##### Survival of small chemical molecules linked to the tripartite [study 10]

When WGA-dextran-gabapentin was used, cross staining with antibodies to gabapentin confirmed preserved antigenicity of the drug after axonal transport of the tripartite, although this method could not itself confirm that the drug was intact or active after transport and release. Dextran-gabapentin with no conjugated WGA did not produce detectable gabapentin antigenicity in the histological sections.

##### Large molecule access to spinal cord from intramuscular injections [study 11]

**Figure 5:**
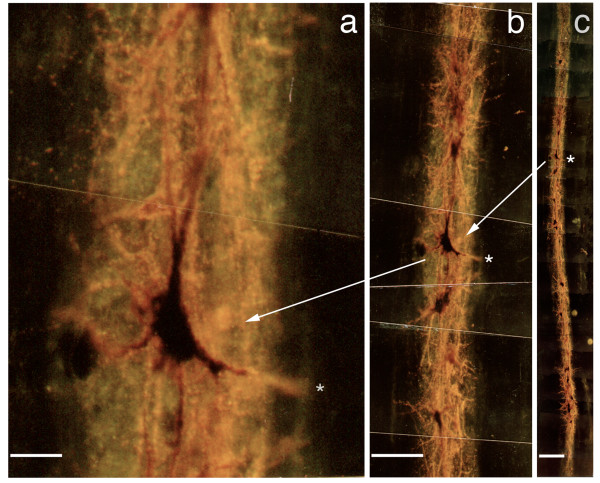
**Axonal transport to spinal cord neurons (longitudinal)**. (**a-c**) Magnified, back illuminated view of an individual motor neuron in an oblique longitudinal section through a portion of the ventral horn of the spinal cord (*Macaca fascicularis*), and seen at lower magnification in figures (**b**) and (**c**). The dark orange material seen inside the cell and filling the cell body and dendritic processes is the product of a chemical reaction carried out by an administered enzyme, horseradish peroxidase. To introduce this exogenous enzyme into the cell, it was conjugated to WGA, an ATF (axonal transport facilitator), then injected into a muscle innervated by the axons which arise from these neuron cell bodies. Scale bars (**a**) = 50 μm, (**b**) = 200 μm, (**c**) = 400 μm.

After small IM injections in muscle, WGA delivered sufficient quantities of functioning HRP enzyme to label numerous motorneurons (Figure 5) in our primate model. Enzymatic activity of the horseradish peroxidase survived any hypothetical lysosomal degradation during transport and yielded product distributed throughout the neuron. Both epaxial and hypaxial muscles proved an effective route to reach spinal cord quickly (several hours) with no apparent difference in delivery of intact enzyme via axons despite the difference in distance of transport. The labeled spinal cord motor neuron pool for these axial muscles had greater longitudinal extent among spinal cord segments than for appendicular muscles. These findings are consistent with previous reports concerning WGA [26] and NGF [27] and their intramuscular introduction [28,29].

The rate of transport appeared to be consistent with time scales predicted by literature values of 30-100 mm/day [30]. Overall the area of spread of injectate in the spinal cord was limited to a few millimeters, although the distribution of the epaxial muscle spinal cord motorneuron pool extended over several centimeters. This appeared to demonstrate that a group of neurons near each other in the spinal cord innervated a group of fibers in epaxial muscle that are close together despite the wider extent of the overall motor neuron pool.

##### Intact transport of targeted nanoparticles by intact axon termini [studies 12, 13, 14, 15]

**Figure 6:**
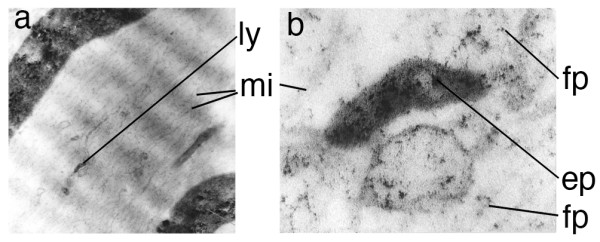
**Intra-axonal location of transported agents**. (**a**) Electron micrograph of rabbit tibial nerve four days after injection of the gastrocnemius muscle with ferrite-WGA tracer. (**b**) magnified view of a vesicle seen in (a) to 195,000×. (ly) lysosomal vesicle, (fp) small particles transporting on microtubules (mi), (ep) larger particles in vesicles.

Electron microscopy showed appropriately sized ferrites within the axon, more than three centimeters from the WGA-dextran-Fe injection site (Figure 6). The appearance of the ferrites in endosomes was similar to what has been seen in studies of similar agents in other tissues [31], but in this case the particles were restricted to the axonal stream. The autoradiographic studies demonstrated sciatic nerve radioactivity and so provided similar evidence that the magnetite containing particles were transported in nerves.

**Figure 7:**
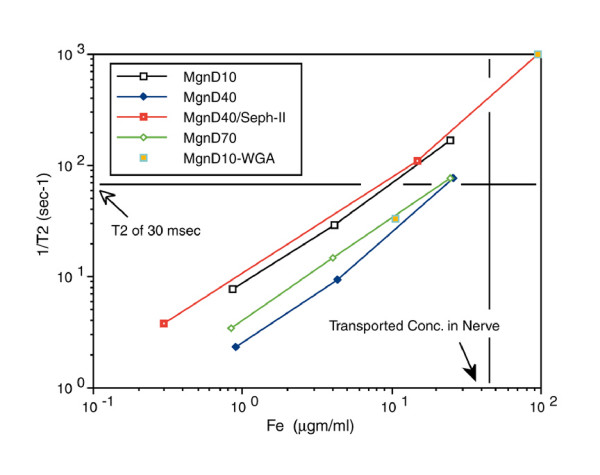
**T2 relaxivity of hydroxide free magnetite preparations**. T2 relaxivity curves for polyacrylamide "tissue" gels polymerized with uniform distributions of various dextran coated magnetite particle preparations. Relaxivity is compared with the concentration of particles in the each gel preparation as assessed by ferrozine assay of iron content after the imaging. 1/T2 was measured in a 4.7Tesla Sisco MR spectrometer. At concentrations comparable to what was achieved in nerve by axonal transport, a T2 below 30 milliseconds would be expected for any of these particle preparations. D10 = 10,000 MW dextran coating, D40 - 40,000 MW, D70 - 70,000 MW, Mgn = magnetite, WGA is wheat germ agglutinin, Seph II - sepharose separated to reduce contamination of magnetite by non-superparamagnetic ferrites.

**Figure 8:**
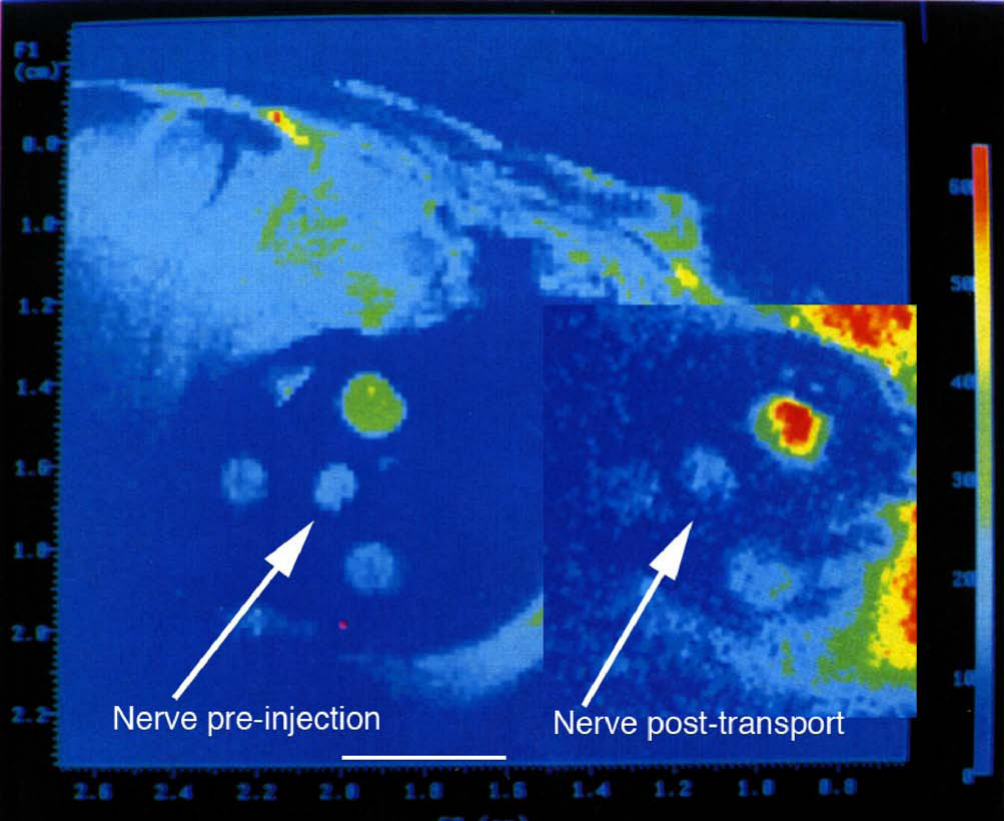
**Microscopic MRI evaluation of sciatic nerve magnetite contrast effect**. Microscopic MRI image of sciatic nerve in calibrating gel chamber in anaesthetized rabbit. The experimental set up is detailed in Figure 19. The wall of the silastic cuff was opened and placed surgically around the sciatic nerve. Serial images with a 2 cm surface coil allows for measurement of the relative T2 intensity of the sciatic nerve by comparison with the T2 of the calibration gels in the three surrounding chambers with the elapse of 8 hours between the pre-injection image and the post-transport image. The injection was carried out immediately following the pre-injection image. The T2 of the sciatic nerve decreases relative to the calibration chambers as axonal transport of the WGA-dextran-magnetite agent progresses. Scale bar is 4 millimeters.

**Figure 9:**
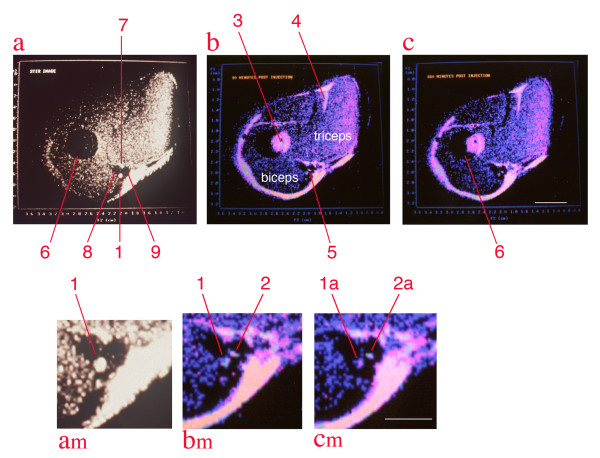
**Median nerve contrast study by solenoid coil high resolution, high field MRI**. All images are from a single image slice of rabbit upper arm. Image (**a**) is collected with a STIR (short tau inversion recovery) sequence which suppresses signal from fat - it reduces the marrow signal (6), and also identifies structure (1) as the median nerve[44], structure (7) as the ulnar nerve, structure (8) as the flow void of he brachial artery and structure 9 as the flow void of the basilic vein. Images (**b**) & (**c**)are colorized spin echo studies obtained at 90 minutes and 360 minutes after injection, respectively. Note that the marrow (3) appears shifted out the humerus (partially overlapped dark circle) by chemical shift effects. The shift at 4.7 Tesla is 1.85 millimeters. Similar shifts are seen at (4), and serve at (5) to leave two bright structures in a gap between triceps and biceps. (**a-m**), (**b-m**), (**c-m**) are magnified views of the space between the biceps and triceps on the medial aspect of the upper arm. Structure (2) is a small amount of fatty tissue that is actually located on the inferior left surface of the brachial artery, but chemical shift has placed its fat image into the midst of the basilic vein. Structure (2) disappears in the STIR image due to fat suppression. Based on this identification, the median nerve (1/1a) is compared to the non-neural structure (2/2a) and is seen to lose intensity in the four and half hour interval between images (**b**) and (**c**) reflecting transport of the WGA-magnetite contrast agent injected in the forearm flexor muscles [24]. The image conspicuity of this structure was measured by multiplying its volume times the intensity in grayscale and this reveals a decrease of 52% in the 270 minute interval. Scale bars are 6 mm for (**a**), (**b**), and (**c**) and 3 mm for (**am**), (**bm**) and (**cm**).

The relaxivity experiments (Figure 7) taken together with distribution studies showed that the concentration of magnetite delivered to the axon by the tripartite was sufficient to affect the T2 relaxation rate of nerve. The observation of a decrease of T2 relaxation time in nerves transporting superparamagnetic nanoparticles in both the micro-MRI nerve channel studies (Figure 8) and in the high resolution MRI experiments (Figure 9) confirmed that the carrier particles were not degraded. Any hydrolysis of the sub-domain sized particles would have eradicated their superparamagnetic effect on T2 relaxation time in nerve as transport progressed. The relaxivity effect far exceeded that which would result from free iron or ferritin at the doses administered.

The WGA-dextran-magnetite experiments in rabbits confirm other reports [32] that particles of 5-15 nm are endocytosed and transported by intact nerve endings. Some studies have suggested that nerve injury is required for the transport of larger particles [33], but this result suggests that intact neurons will indeed transport large (up to 15 nm) multi-molecular aggregates or "transport particles" after intramuscular injection when the particles are well solvated.

### C. Targeting and pharmacological efficacy (aspect C)

#### Clinical target access (project VI)

##### Targeted Access to Clinically Relevant Neuronal Sub-populations [studies 16, 17, 18, 19]

**Figure 10:**
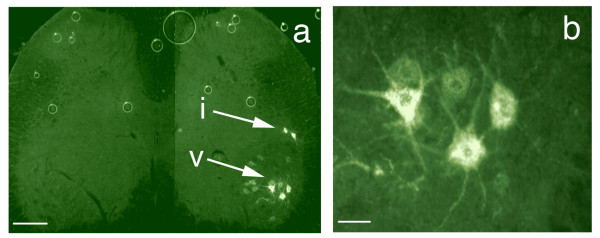
**Axonal transport to spinal motor neurons and autonomic neurons**. (**a**) Section of rat spinal cord showing retrogradely transported WGA-FITC in the motor neuron cell bodies (v) and in cells in the autonomic intermediolateral cell column (i). (**b**) magnified view of motor neurons seen in (**a**). Scale bars (**a**)= 120 μm, (**b**) = 30 μm.

**Figure 11:**
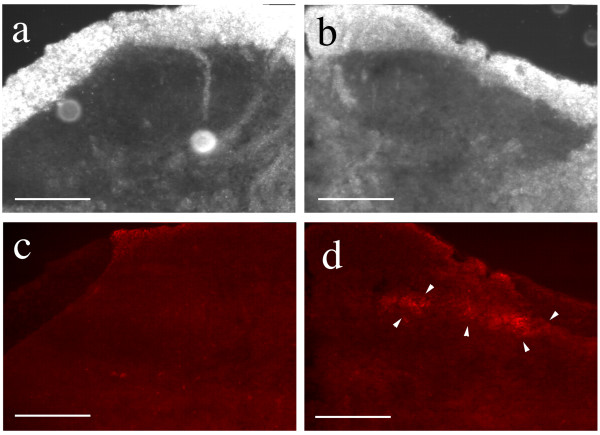
**Dorsal root entry zone access by transported agents**. Section of spinal cord showing retrogradely transported NGF-TR (Nerve Growth Factor - Texas Red) in the dorsal horn. (**a+b**) The ipsilateral and contralateral dorsal horn viewed using darkfield microscopy. (**c+d**) The same fields as in **a+b** viewed using fluorescence microscopy. The arrow heads delineate the area of DREZ lamina I and II where the proximal axons of the dorsal root ganglia cells terminate on nociceptor neurons. Scale bars = 120 μm.

**Figure 12:**
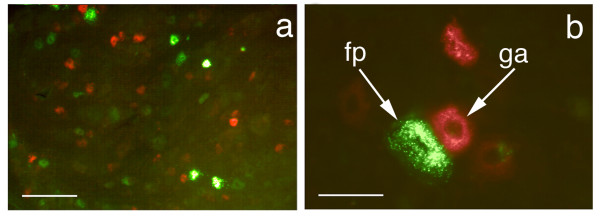
**Axonal transport to dorsal root ganglion neurons**. (**a**) Delivery to rat dorsal root ganglion cells from different peripheral sources. Section of L4 dorsal root ganglia showing retrogradely transported FITC (green) injected intra-muscular and TRITC (red) injected intra-plantar, in the sensory neuron cell bodies (fp - footpad injection, ga - gastrocnemius injection) (**b**) higher resolution view. Scale bars (**a**) = 170 μm, (**b**) = 45 μm.

**Figure 13:**
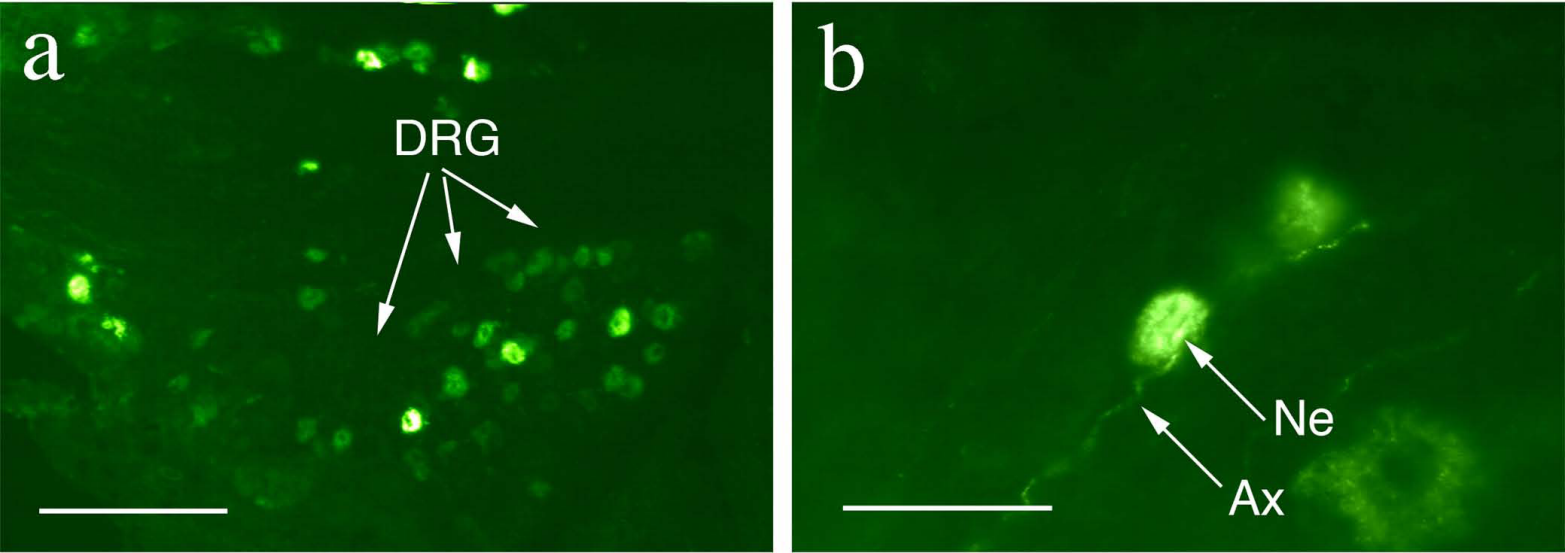
**WGA-dex-FITC transport to dorsal root ganglion cells**. (**a**) Section of L4 dorsal root ganglia showing retrogradely transported FITC in the sensory neuron cell bodies. (**b**) The FITC can be seen in sensory dendrites arriving at the cell. Ax - axon, DRG - dorsal root ganglion, Ne - neuron cell body. Scale bars (**a**) = 150 μm, (**b**) = 80 μm.

Intramuscular injection of the tripartite WGA-dextran-FITC produced labeling of alpha motor neurons in the ventral horn and autonomic neurons in the intermediolateral cell column (Figure 10). We also observed good filling of proximal sensory neuron processes (axons) in the dorsal root entry zone and in lamina I and II of the dorsal horn of the spinal cord (Figure 11). Injection of foot pad and multiple hind limb muscle each resulted in labeling of less than 50% of ganglion cells, but injection of both muscle and skin resulted in filling of nearly 90% of dorsal root ganglion (DRG) cells (Figures 12 &13). In clinical use, the objective will typically be to reach specific sub-populations rather than filling an entire ganglion *per se*, so these results support the expectation that a large fraction of cells in a e.g. a subpopulation innervating a single muscle or patch of skin can readily be reached.

**Figure 14:**
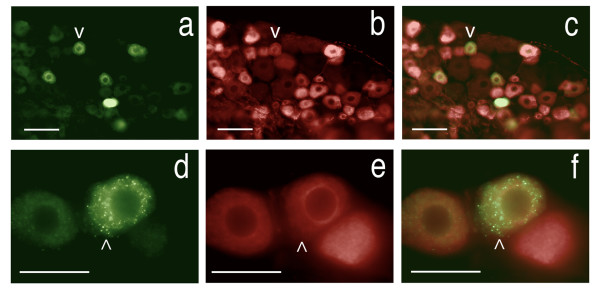
**Demonstration of transport to C-type nociceptor cells in dorsal root ganglion**. Section of rat L5 dorsal root ganglia showing retrogradely transported FITC combined with immunohistochemistry for the specific C-fiber nociceptor marker, Peripherin. (**a**) - Retrogradely transported FITC, (**b**) - the same field as in (**a**) showing cells that are immuno-positive for peripherin, (**c**) - overlay of (**a**) and (**b**) showing FITC is present in the nociceptors (v - same neuron in **a**, **b**, &**c**). There are 26 cells seen with FITC in (**a**) versus 59 cells with peripherin in (**b**) & (**c**). (**d**) - Two sensory neurons containing retrogradely transported FITC, a third neuron is unlabeled, (**e**) - the same field as in (**d**) showing that all three neurons are positive for Peripherin, (**f**) - overlay of (**d**) and (**e**) (^ - same neuron in **d**, **e**, &**f**). Scale bars (**a**-**c**) = 150 μm, (**d**-**f**) = 40 μm.

Cross staining with an antibody to peripherin showed that many of the DRG sensory neurons that were accessed were C-fiber nociceptor cells (Figure 14). This helps confirm that this method of delivery does reach a selected subset of nociceptors that correlates specifically with the selected site of injection. The ATF played a major role since no detectable fluorescence was observed when dextran-FITC without ATF was administered in these experiments.

#### Distinctive pattern of distribution relative to trans-vascular (project VII)

##### Unique distribution with high fraction of drug reaching neuronal targets [study 20]

**Figure 15:**
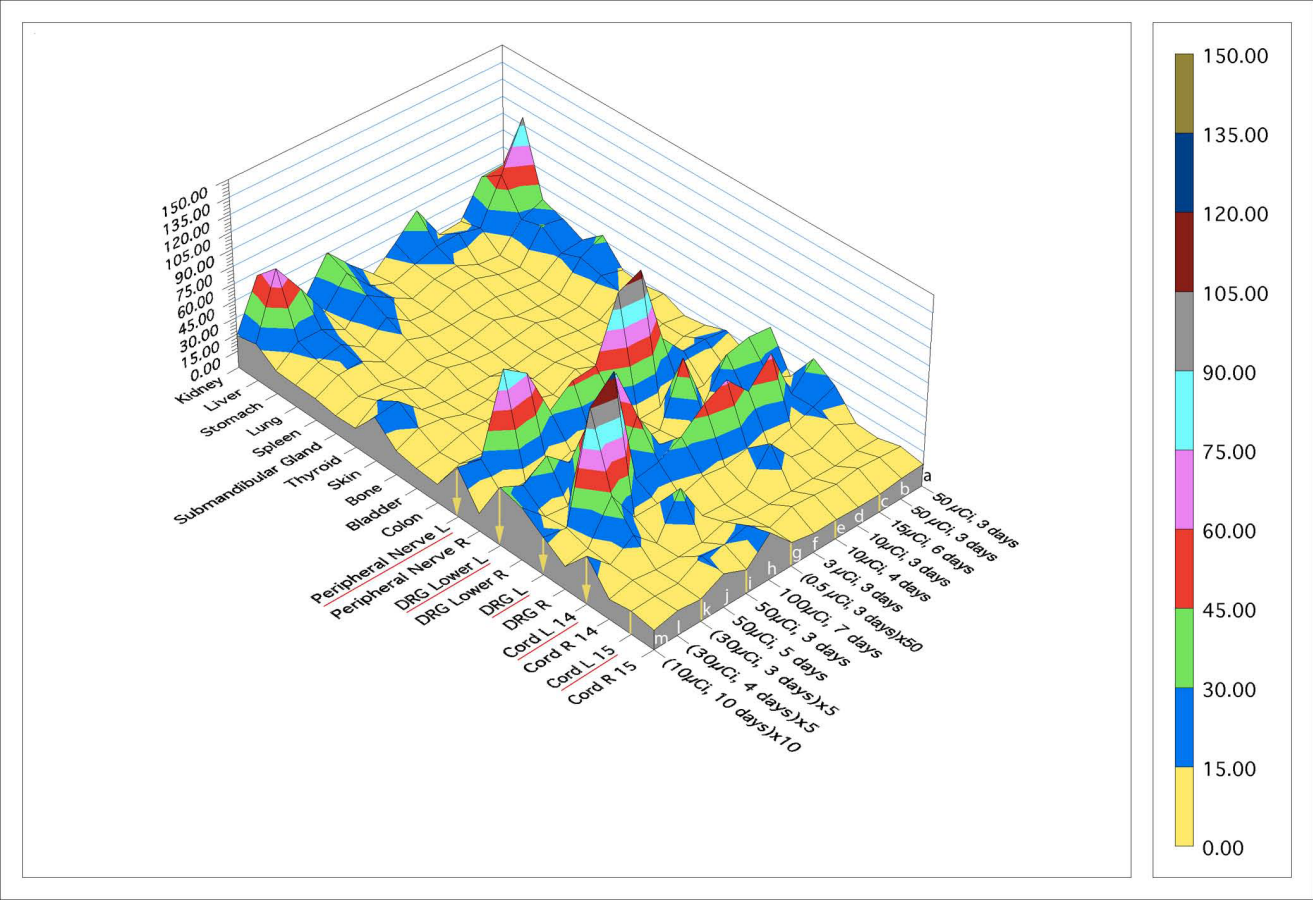
**Tissue distribution data for intramuscular [^125^I]-WGA**. Results are standardized by dividing all results for a given animal by the cpm/mg in blood for that animal. This results in a dimensionless figure proportional to concentration in tissue relative to concentration in blood for each tissue in each animal. Data are arrayed in chronological experimental sequence order as follows: (***a***) and (***b***) - incision with suture closure and 3 day survival; (***c***) - similar to (**a**) & (**b**) but longer survival time; (***d***) - superglue seal on the incision, animals kept in a metabolic cage; (***e***), (***f***), and (***g***) - the [^125^I]-WGA was concentrated into one tenth the volume; (***h***) - large dose with 20 injection locations of 0.5 microliters each and longer survival; (***i***) and (***j***) - ten small injections; (***k***) - 10 gm-cm spinal cord injury; (***l***)- 25 gm-cm spinal cord injury; (***m***) - very long survival of 10 days (multiplied by 10 to emphasize very low retention).

In the whole body distribution studies with small calf muscle injections, the concentration of [^125^I]-WGA detected in relevant ipsilateral peripheral nerve and dorsal root ganglia reached six times systemic concentrations (Figure 15). The detected concentration in relevant nerve and ganglia (the sciatic nerve and DRGs in continuity with the sciatic nerve) demonstrated saturability and transport time consistent with other reports [34]. Activity in spinal cord was less than in nerve or DRG but did reach twice systemic concentrations when higher concentrations of injectate were used.

There was no significant activity detected in contralateral nerve and DRGs nor in ipsilateral nerves, ganglia, or spinal cord related to cervical, thoracic, or higher lumbar segments. The greatest activities detected in other tissues were in liver and kidney but these apparently reflected metabolized iodine lost from the compound.

NGF showed less transport to spinal cord than was seen for WGA consistent with selective uptake of NGF by sensory and autonomic nerves. Detection of activity due to [^59^Fe]-WGA-dextran at other systemic sites was even less than for WGA or NGF alone.

Considerable amounts of drug remained in the axon itself at the time points sampled. It is known that para-nodal complexes of Schwann cells at the Nodes of Ranvier can endocytose materials from the axoplasm [35]. This may account for a peri-axonal depot effect with drug being cleared to the para-nodal complexes and then subsequently being re-released. Further studies will be needed to clarify this issue.

##### Site of injection retains high amount of injectate [study 21]

The regional view provided by the [^131^I]-WGA gamma camera studies demonstrates that the total amount of the injectate remaining at the site of injection can be large when compared to the amount in the small volume of the nerve. In part, this reflects the fact that WGA adheres to muscle cell membranes [36] as well as being taken up by adsorptive endocytosis at nerve termini. This also explains a depot effect whereby additional amounts of the compound continue to be introduced into the axonal stream over a period of several days.

##### Pharmacologically efficacious drug amounts were delivered [study 22]

The tripartite assemblage with [^14^C]-labeled gabapentin produced activity levels for gabapentin in ipsilateral neurons of more than 600 times greater than background while counts remained at background levels in contralateral neurons. This demonstrated the localizing effect of injection of polymer-bound drug conjugated to an axonal transport facilitator, and also allowed us to estimate the drug concentration achieved based on the specific activity of the [^14^C]-labeled gabapentin.

#### Pharmacologic effects of ATF mediated delivery not obtainable by trans-vascular agents (project VIII)

##### Prolonged suppression of hyperalgesia [study 23]

**Figure 16:**
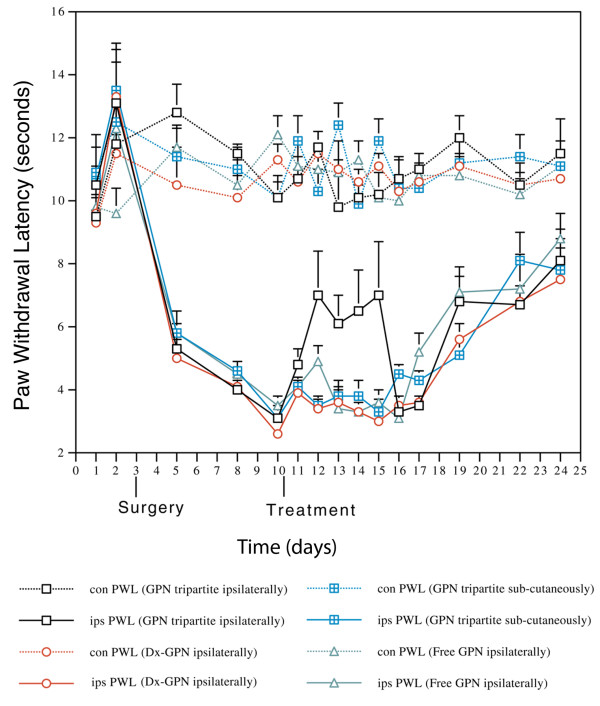
**Effect of gabapentin delivered by intraneural vehicle**. The effect of Gabapentin tripartite administration, to mono-neuropathic animals, on thermal nociceptive threshold (con, contralateral; ips, ipsilateral; GPN, Gabapentin). Results are the mean ± SEM (n = 6). The difference between the ipsilateral and contralateral side is significant from 4 days post surgery to the end of the experiment in all treatment groups. Treatment with Gabapentin tripartite to the injured limb caused a significant elevation in paw withdrawal latency which was evident within 2 days and lasted for a further 4 days, after which the latency returned to that of the other groups (e.g. day 12, P = 0.0049 between Dx-GPN and GPN tripartite ipsilaterally). This effect was not observed in any other group.

In the hyperalgesia experiments a single injection with the tripartite agent reduced the hyperalgesia (p < 0.05 in eight out of the nine comparisons of treatment versus control) for at least four days after injection (Figure 16). The total dose was 0.375 mg/kg using the novel intraneural transport method compared to 120 mg/kg total dose orally for four days of the usual human oral dosing. Prolonged efficacy may have been due to a depot effect in muscle, slow redistribution from axon and Schwann cells to nerve cell body, slow clearance from inside the nerve, or preemptive blocking of newly produced receptor molecules en route from ribosome to cell surface.

## Discussion

We have demonstrated through a comprehensive series of chemical, radiological *in vitro*, and *in vivo *biological studies that axonal transport is a valid route for drug delivery when a tripartite (ATF-polymer-drug) complex is used. We have used these studies to exemplify *in vivo *a prolonged pharmacologically effective delivery of an analgesic molecule leading to sustained significant response in a standard nociceptive model.

Routine oral delivery of gabapentin results in clinical efficacy for pain relief when blood levels reach 100 μM. Gabapentin not only crosses the blood brain barrier but is subject to some active concentration [37,38]. It is reasonable to hypothesize, therefore, that clinically efficacious axonal transport for intraneural drug delivery would need to produce similar concentrations near the internal cell surface of accessed neurons to achieve similar pharmacological efficacy.

In this model, histological estimates of the ratio between the estimated intracellular volume of the accessed neurons (InCvol) and the actual volume of the entire dorsal root ganglion (DRGvol) provide a correction factor (InCvol/DRGvol) that allows for conversion of total DRG concentration into estimated intracellular concentration in the accessed neurons.

When these considerations were employed, our counts of delivered [^14^C] suggested delivery of gabapentin at intracellular doses equal to or greater than 100 μM. These *in vivo *[^14^C]-gabapentin studies therefore showed that this technology can deliver drugs to target neurons at pharmacologically efficacious doses using 1/300^th ^of the oral dose that would be required to achieve the same intracellular concentration in target cells.

In addition, this system resulted in a ten fold increase in effective half life relative to oral administration. This appears to be because of the unique dynamics of this delivery mechanism. This involves both a muscle injection site depot effect for the large tripartite complexes and a prolonged stream of delivery to the neuron from the axon and its associated paranodal complexes.

Use of the tripartite vehicle made it possible to use a selected axonal transport facilitator to cause transport of large numbers of drug molecules and to recover the pharmaceutical effect of the drug after delivery to its target. By varying the polymer backbone length and by use of various linker chemistries, it is possible to accommodate therapeutic molecules with a wide variety of different sizes and physico-chemical properties.

In this set of experiments, neither systemic delivery of the agent by a subcutaneous route nor administration of free drug alone to the affected limb had any significant therapeutic effect. Although dextran can itself weakly promote axonal transport [39], the use of dextran/drug with no ATF showed only limited effect in occasional animals and was in no case statistically significant. This takes advantage of the greatly improved efficiency of targeted adsorptive endocytosis - the tripartite complexes specifically adhere to markers exposed on the external surface of the axon terminus at sites destined to be drawn into the interior of transport vesicles. These markers can be used in the phage display system to develop new purely synthetic, non-viral ATFs to offer selectivity for target neuron sub-types. The system is also applicable for introducing DNA to the neuronal nucleus without the risks of viral transfection systems.

It had not been clear in the past whether gabapentin acts inside nerve cells or on the external cell surface [40-42]. Further, the conjugation reaction and subsequent release of the gabapentin might have converted it to a chemically distinct metabolite or residue. The high efficacy of this intraneurally delivered agent constructed with gabapentin demonstrates for the first time that delivery of a clinically efficacious small molecule therapeutic agent from inside the cell via an intraneural route can achieve the same pharmacological effect as delivery from outside the cell via a systemic route. This also corroborates recent evidence that the site of action of gabapentin is indeed intracellular [43].

One of the functions of retrograde axonal transport is to allow the cell body to sample conditions at the distant axon terminus in order to obtain information on requirements for replacement organelles, proteins, and small molecules. Excessive hydrolysis in lysosomal transport vesicles would interfere with this sampling process, so the preservation of therapeutic molecules during axonal transport in vesicles is consistent with this function.

Chronic use of axonal transport agents raises the issue of specialized toxic effects associated with the delivery system. In general, the amount of drug being delivered to the axonal intracellular space will be similar to normal therapeutic delivery and will be handled by the same clearance methods that the cell usually relies on. The ATF will be delivered in amounts that are physiologic and will present no special accumulation risk although there may be unintended direct intracellular signaling effects from some ATFs that would need to be identified. The dextran or other polymer linker could be subject to accumulation, however it must be kept in mind that the axon is constantly ingesting extracellular fluid as a means of sampling the environment of the axon terminus for presentation to the regulatory apparatus in the cell nucleus after transport. The cargos delivered by the endosome/lysosome system physiologically must be degraded and removed from the cell. We did not observe any apparent toxicity upon histological analysis of the CNS or axonal structures in any of these experiments, however, this issue would be need to be carefully assessed in the course of development of any pharmaceutical for axonal delivery.

## Conclusions

These studies demonstrate that intraneural delivery of pharmaceutical agents as part of a tripartite complex (axonal transport facilitator + polymer + multiple conjugated drug molecules) results in a unique distribution in which high concentrations reach targeted CNS, autonomic and peripheral nerve targets. The resulting concentration in non-targeted CNS and systemic tissues is several orders of magnitude lower than the concentration in targeted CNS and PNS tissues. This effect can be achieved using a well tolerated and non-invasive clinically applicable administration route - intramuscular and intradermal injection.

Whilst there are additional issues to resolve before axonal transport based medications come into regular clinical use, their eventual development now seems realistic. Previously, the relatively small number of 'uptake events' per neuron for molecules like NGF has limited their applicability for intraneural drug delivery. This work demonstrates that a tripartite complex with a polymer linker carrying large numbers of drug molecules can amplify the pharmacological effect of each uptake event by at least two orders of magnitude. Amplification by an additional order of magnitude through alternative conjugation schemes appears to be readily achievable.

The delivery to DRG neurons including nociceptors as well as to motor column neurons has positive implications for the development of medications for pain, muscle spasm, neuroprotection and anti-viral treatment. We anticipate that intraneural pain medication will have a significant impact on the management of pain after surgery and in the treatment of patients suffering from intractable chronic pain unresponsive to existing pain medications. Some efficacious agents whose use is limited by systemic toxicity may be safely and effectively delivered by an axonal transport delivery vehicle. It is likely that anti-viral, neuroprotectant and anti-spasmodic agents (small molecule, peptide, or nucleic acid) can also be delivered to important locations in the nervous system in this manner. The application of this new class of tripartite intraneural pharmacologic vehicles also provides a novel tool for the investigation of a number of aspects of basic neurobiology.

## Methods

These are summarized in Table 1.

### Synthesis and Stability (aspect A)

#### Chemical Methodology: Syntheses of various tripartites (project I)

##### Assembly and loading of tripartite drug carriers [study 1]

We constructed a tripartite drug delivery vehicle using carboxyl linkages to bind multiple molecules of the selected drug to each 70,000 MW dextran molecule, then conjugated WGA to the dextran to act as axonal transport facilitator (ATF) (Figure 1). Efficiency of loading of the polymers by drug or label molecules was assessed with nuclear magnetic resonance (NMR) spectroscopy or by high pressure liquid chromatography (HPLC) with mass spectrometry. Iterative variations of synthetic techniques were carried out to achieve progressively greater levels of drug loading with preservation of the targeting affinity of the ATF portion of the complex.

*Carboxy modified dextran (standard loading) (protocol 1a)*

Dextran (MW 70,000; Sigma) (324 mg) was dissolved with stirring in water (30 ml) and the solution made basic by the addition of 2 M potassium hydroxide (50 μl). Cyanogen bromide (424 mg) was added and the solution treated with 2 M potassium hydroxide (50 μl) at 15 minute intervals for the next 2 hours. After stirring for a further 2 hours, more cyanogen bromide (424 mg) was added and the solution treated with 2 M potassium hydroxide (50 μl) every 15 minutes for a further hour. Thesolution was then stirred at room temperature overnight. 6-Aminohexanoic acid (5.24 g) was added and the solution adjusted to pH 11 by the addition of 10 M potassium hydroxide. The solution was then stirred at room temperature for two days and the solution adjusted to pH 2 by the addition of hydrochloric acid. After filtration through a 0.45 μm membrane the solution was concentrated with a stirred cell (membrane MWCO 30,000) and the concentrate washed similarly four times with water. The resulting solution was lyophilized to afford a colorless solid, 272 mg. ^1^H NMR analysis (400 MHz, D_2_O) suggested a carboxy loading of 11-12%.

*Carboxy modified dextran (Aminohexanoic acid) - Upgraded loading (protocol 1b)*

Dextran (MW 70,000; Sigma) (162 mg) was dissolved with stirring in water (30 ml) and the solution made basic by the addition of 2 M potassium hydroxide (50 μl). Cyanogen bromide (212 mg) was added and the solution treated with 2 M potassium hydroxide (50 μl) at 15 minute intervals for the next 2 hours. A second portion of cyanogen bromide (212 mg) was added and the solution treated with 2 M potassium hydroxide (50 μl) every 15 minutes for a further hour. 6-Aminohexanoic acid (2.62 g) was added and the solution adjusted to pH 10.5 by the addition of 2 M potassium hydroxide. The solution was then stirred at room temperature for two hours and then adjusted to pH 2 by the addition of hydrochloric acid. After filtration through a 0.45 μm membrane the solution was concentrated with a stirred cell (membrane MWCO 30,000) and the concentrate washed similarly four times with water. The resulting solution was lyophilized to afford a colorless solid, 180 mg. ^1^H NMR analysis (400 MHz, D_2_O) at 70°C suggested a carboxy loading of 30%.

*Carboxy modified dextran (extended chain length linker) (L-Lysine diacetic) (protocol 1c)*

Dextran (MW 70,000; Sigma) (81 mg) was dissolved with stirring in water (15 ml) and the solution made basic by the addition of 2 M potassium hydroxide (50 μl). Cyanogen bromide (106 mg) was added and the solution treated with 2 M potassium hydroxide (50 μl) at 15 minute intervals for the next 2 hours. A second portion of cyanogen bromide (106 mg) was added and the solution treated with 2 M potassium hydroxide (50 μl) every 15 minutes for a further hour. L-Lysine-*N*_*a*_, *N*_*a*_-diacetic acid (724 mg) was added and the solution adjusted to pH 10.5 by the addition of 2 M potassium hydroxide. The solution was then stirred at room temperature overnight and then adjusted to pH2 by the addition of hydrochloric acid. After filtration through a 0.45 μm membrane the solution was concentrated with a stirred cell (membrane MWCO 30,000) and the concentrate washed similarly four times with water. The resulting solution was lyophilized to afford a colorless solid, 110 mg. ^1^H NMR analysis (400 MHz, D_2_O) at 70°C suggested a carboxy loading of 27% (81% with respect to carboxylate).

*WGA-carboxydextran conjugate (protocol 1d)*

Carboxy modified dextran (80 mg) was dissolved in water (20 ml) and the solution made basic by the addition of 2 M potassium hydroxide (50 μl). Cyanogen bromide (212 mg) was added and the solution treated with 2 M potassium hydroxide (50 μl) every hour for the next 6 hours. The solution was stirred at room temperature overnight and then concentrated with a stirred cell (membrane MWCO 30,000) and the concentrate similarly washed four times with water. After concentration to 6 ml, WGA (3 mg) in 100 mM CaCl_2_/MnCl_2 _(4 ml) was added and the solution stirred overnight. The mixture was applied to an N-acetylglucosamine (NAcGlu) affinity matrix column and the column washed with water. Elution with 800 mM NAcGlu (10 ml), stirred cell purification (MWCO 30,000) with four water washes and lyophilization afforded bipartite as a colorless solid, 2 mg.

*WGA-carboxydextran-valacyclovir conjugate (protocol 1e)*

WGA-carboxydextran conjugate (2 mg) was dissolved in 100 mM CaCl_2_/MnCl_2 _(1.5 ml). Valacyclovir (20 mg) and EDC (1-Ethyl-3- [3-dimethylaminopropyl] carbodiimide) hydrochloride (20 mg) were added and the solution stirred at room temperature overnight. Filtration through a 0.45 μm membrane, concentration with a stirred cell (membrane MWCO 30,000) with four water washes and lyophilization afforded tripartite as a colorless solid, 1.5 mg.

*Carboxydextran-gabapentin conjugate (protocol 1f)*

Carboxy modified dextran (162 mg) was dissolved in water (15 ml). Gabapentin (200 mg) and EDC hydrochloride (575 mg) were added and the solution stirred at room temperature overnight. Filtration through a 0.45 μm membrane, concentration with a stirred cell (membrane MWCO 30,000) with four water washes and lyophilization afforded bipartite as a colorless solid, 145 mg.

*g. WGA-carboxydextran-gabapentin conjugate (protocol 1g)*

Carboxydextran-gabapentin conjugate (25 mg) was dissolved in water (10 ml). Cyanogen bromide (30 mg) was added and the solution treated with 2 M potassium hydroxide (10 μl) every hour for the next 6 hours. The solution was stirred at room temperature overnight and then concentrated with a stirred cell (membrane MWCO 30,000) and the concentrate similarly washed four times with water. After concentration to 3 ml, WGA (1 mg) in 100 mM CaCl_2_/MnCl_2 _(2 ml) was added and the solution stirred overnight. The mixture was applied to an N-acetylglucosamine (NAcGlu) affinity matrix column and the column washed with water. Elution with 800 mM NAcGlu (10 ml), stirred cell purification (MWCO 30,000) with four water washes and lyophilization afforded tripartite as a colorless solid, 4.5 mg.

##### Synthesis of superparamagnetic hydroxide free ferrite particulate carriers [study 2]

Assessment of size limits included synthesis of hydroxide-free (non-reactive) superparamagnetic magnetite particles coated with dextran conjugated to WGA. These were capable of serving as synthetic vectors for gene delivery and capable of identification by electron microscopy, [^59^Fe] radiolabel studies, as well as having sufficient relaxivity contrast effect to be detectable by microscopic MRI (magnetic resonance imaging) and MR Neurographic techniques [44,45].

*Precipitation of water soluble superparamagnetic particles [protocol 2a]*

Using double distilled water (not de-ionized) to make up the reaction mixture the following steps were conducted: 1.5 ml of 33% NH_3 _was added to 4.5 ml of hot dH_2_O (to make up 7.5% NH_4_OH) and brought to 60°C standing in a capped universal tube in a water bath.

1.25 gm Dextran (MW 10,000) was dissolved in 2.0 ml of ddH_2_O and 225 mg FeCl_3_.6H_2_O dissolved in the dextran solution. 100 mg FeCl_2_.4H_2_O is dissolved in the Fe3/dextran solution and the mixture kept in a 60°C water bath for two minutes before incremental addition of 6 ml of hot 7.5% NH_3 _solution (60°C). The product was left to stand in the 60°C water bath for fifteen minutes.

The reaction product (dextran-coated ferrites) was centrifuged three times at 1,000 g for 10 minutes and any precipitate discarded each time. The supernatant was applied to PD-10 columns (Pharmacia) equilibrated with 0.1 M NaAcetate buffer, pH 6.8 with 5 mM EDTA (ethylenediaminetetraacetic acid).

The black eluted fraction was diluted 1:3 with EDTA/Acetate buffer then concentrated to one-tenth the initial volume with Amicon Centriprep-100 ultrafilters. The retentate was diluted 1:10 with EDTA/Acetate buffer then concentrated to a volume of 1.5 ml with the Centriprep-100 ultrafilters.

*Periodate conjugation of targeting agent [protocol 2b]*

0.30 ml of 20 mM NaIO_4 _was added to the dextran ferrite solution (approx. 1.5 ml) while stirring then gently tumbled for 60 minutes at room temperature in the dark. At the end of the 60-minute periodate incubation, the reaction was terminated by applying the reaction mixture to the PD-10 columns equilibrated with 20 mM borate buffer (pH 8.5).

An active site blocking solution was prepared using 100 mM MnCl_2_/CaCl_2 _for WGA binding reactions to protect the WGA binding site during the conjugation reaction.

10 mg of the ATF protein was dissolved in 500 ml of 20 mM Na borate buffer, pH 8.5 at room temperature. 10 ml of the blocking solution was added to the protein/borate solution then 2.0 ml of oxidized magnetite dextran mixed with 500 ml of the protein/borate solution. 20 ml of the blocking solution was added into the 2.5 ml protein-dextran-magnetite mixture and mixed well, then incubated for 6 to 18 hours at room temperature with shaking.

After the incubation, 100 ml of 0.5 M glycine was added to the reaction mixture and incubated for 2 hours. 250 ml of 0.25 M NaBH_4 _was added to the magnetite-dextran-protein solution and allow to stand for 60 minutes, shaking periodically to release H_2 _gas. At the end of the incubation the reaction mixture was passed through PD-10 columns equilibrated with 20 mM HEPES buffer, pH 7.4. The eluant was diluted 1:5 with HEPES buffer then concentrated with Centriprep-100 ultrafilters.

*Affinity purification [protocol 2c]*

An affinity purification step is optional and detail is given for use with a WGA (lectin) targeting protein. The final retentate was applied to affinity columns (N-Acetyl-Glucosamine) (20 mM HEPES buffer), washed with HEPES, then specifically eluted with 1 M NAcGlu in HEPES buffer, pH 7.4. The specific eluant was passed through PD-10 columns equilibrated with HEPES to remove NAcGlu, Mn and Ca.

The desalted output was then diluted to a volume of 24 ml with HEPES buffer and concentrated with Centriprep-100 concentrators. The final retentate was sterilized by spinning at 500 g for one hour in 0.22 mm centrifugal microfilters. The purified, sterilized synthetic vector particles were stored at 4°C for use within one to two weeks. (not be frozen or lyophilized).

*DNA adhesion [protocol 2d]*

The methodology for carrying out and demonstrating DNA adhesion and uptake with these particles is detailed elsewhere [46].

*Relaxivity assessment [protocol 2e]*

Phosphate buffered polyacrylamide gel phantoms (C% = 2.7, T% = 11) were cast in test tubes after mixing with various concentrations of WGA-dex-mag using various different dextran lengths. The gels were used to simulate animal tissue for MRI relaxivity testing as described previously [47]. Gel tubes were placed in solenoid coils for T2 measurements in a 4.7 Tesla SISCO MRI spectrometer. Subsequently, the gels were subjected to ferrozine iron assays to verify the iron concentrations [48]. This resulted in relaxivity data demonstrating the concentration needed to reduce T2 below 30 msec - which would be readily visible relative to many other tissues with longer T2 (Figure 7).

#### Intracellular release from tripartite and drug activation in cell culture (project II)

##### Uptake and intracellular drug release in a Valacyclovir BHK plaque assay [study 3]

*In vitro *comparison of the antiviral efficacy of free valacyclovir, valacyclovir linked to dextran and WGA-dextran-valacyclovir was tested in a plaque reduction assay to evaluate the impact of targeting molecules on endocytosis of large drug carrying complexes and to assess potential efficacy of drug molecules released from the tripartite complex after cellular endocytosis in a Herpes simplex virus/BHK (baby hamster kidney) cell model.

Plaque reduction assays were designed via modification of methods previously described [49,50]. The conjugation strategy for the valacyclovir prodrug to the dextran backbone assured that hydrolysis of the molecular link would be required to release drug and that the hydrolysis would release active acyclovir rather than the inactive prodrug [51].

The virus used throughout these studies was HSV-1 strain SC16 (originally isolated from a clinical case of *Herpes labialis*). This strain has been extensively characterized in mice and has been previously use for studying antiviral compounds. Viral working stocks were produced at a low multiplicity of infection in BHK-21 cells (baby hamster kidney cells) and stored in small aliquots at -70°C. Maintenance of BHK-21 cells was performed using standard procedures.

The conjugate stoichiometries were approximately Dx(70):Val = 1:50; WGA:Dx(70):Val = 2:1:50. In terms of valacyclovir (w/w), the free Val:Dx-Val:WGA-Dx-Val ratio is assumed to be 1:0.1:0.08 (i.e. correcting for vehicle mass). Thus, 1 mg (free) = 10 mg (di-partite) = 12.8 mg (tri-partite). Therefore, stock solutions of 1 mg/ml (free drug), 10 mg/ml (di-partite), 12.8 mg/ml (tri-partite) were required to ensure equivalent valacyclovir added for a given volume addition.

In initial range finding plaque reduction tests, the free drug was approximately 10-fold more potent as an anti-viral than the Dx-Val conjugate and a more suitable range of between 0-10 μg was chosen for the free drug assay, whilst a range between 0-100 μg was required for di-partite. Testing the tri-partite compound, initially a range of 0-100 μg valacyclovir was chosen. However, this was found to be overload (i.e. no plaques observed at the first data point corresponding to 5 μg val). This suggested that the IC50 for the tri-partite would be about 2.5 μg.

To ensure the addition of identical volumes in each assay a 10-fold dilution of the free drug stock was made to give 0.1 mg/ml.

Finally, volumes of between 0-100 μl anti-viral solution (equivalent to 0-100 μg valacyclovir in non-ATF conjugated form or 0-10 μg in free form or tripartite form) were added to wells of BHK cells infected with HSV-1 and plaque numbers counted (in replicates of 6) following a three day incubation.

### Interaction with Axon Terminals & Intra-Axonal Environment (aspect B)

#### Effects of polymer, linker and drug (project III)

We used cultured dissociated sympathetic ganglion neurons to evaluate the effect of polymer size, of molecular charge and of chemical side groups that varied the hydrophilicity of the molecular complex upon efficacy of uptake by neurons in open cultures.

##### Size Dextran-FITC [study 4]

We synthesised a series of dextran-based FITC-containing polymers coupled to WGA. In an initial series, we evaluated the effects of differences in dextran size on uptake by cultured sympathetic ganglia. For the size trials, we incubated the ganglia cultures with the tripartite for varying lengths of time, then washed the cultures and examined them using fluorescence and also backlit fluorescence microscopy.

##### Charge Effects on Dextran-FITC Neuronal Uptake [study 5]

Each material was based on dextran-70. Dextran-70 was used to best illustrate the effects of the modification rather than using dextran-10 which had more efficient uptake in its native state. The charge of the materials was created by activating the sugar monomers and coupling with either a carboxyl terminated linker or an amine terminated linker. The neutral version was achieved by coupling a 50:50 mix of carboxyl and amine linkers. WGA and FITC was coupled as well. The various agents were incubated with cultured sympathetic ganglia after which the media were washed and evaluation was of uptake was carried out by backlit fluorescence microscopy capable of viewing neurite projections with or without the presence of transported agent.

##### Acylation effect [study 6]

We carried out various degrees of acylation of FITC labeled dextran followed by conjugation with WGA in order to test the effects of increased hydrophobicity on uptake in cultured sympathetic ganglia. Once again dextran-70 was used to best illustrate the effects of the modification rather than using dextran-10 which had more efficient uptake in its native state. FITC labeled, WGA-conjugated dextran-70 with no acylation was used as a control.

#### Effects of Axonal Transport Facilitator (ATF) (project IV)

##### Campenot Chamber - Comparison of physiologic vs non-physiologic ATF [study 7]

**Figure 17:**
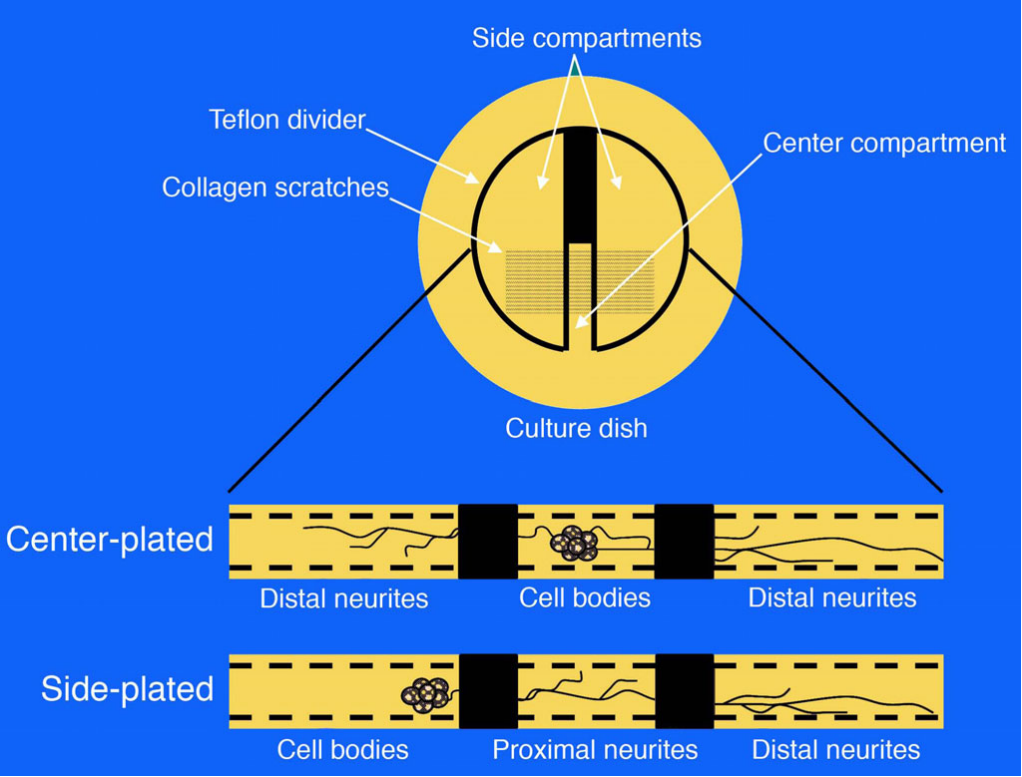
**Campenot chamber principles for compartmented cultures**. The culture dish is coated with collagen, and then the substratum is scarified with a pin rake. A shaped Teflon gasket with silicon grease on its bottom edges is placed in the chamber over the scarified tracks. Dissociated mouse superior cervical ganglion neurons are then plated in the central chamber. Neurite outgrowths are then confirmed as they project into the adjacent chambers.

**Figure 18:**
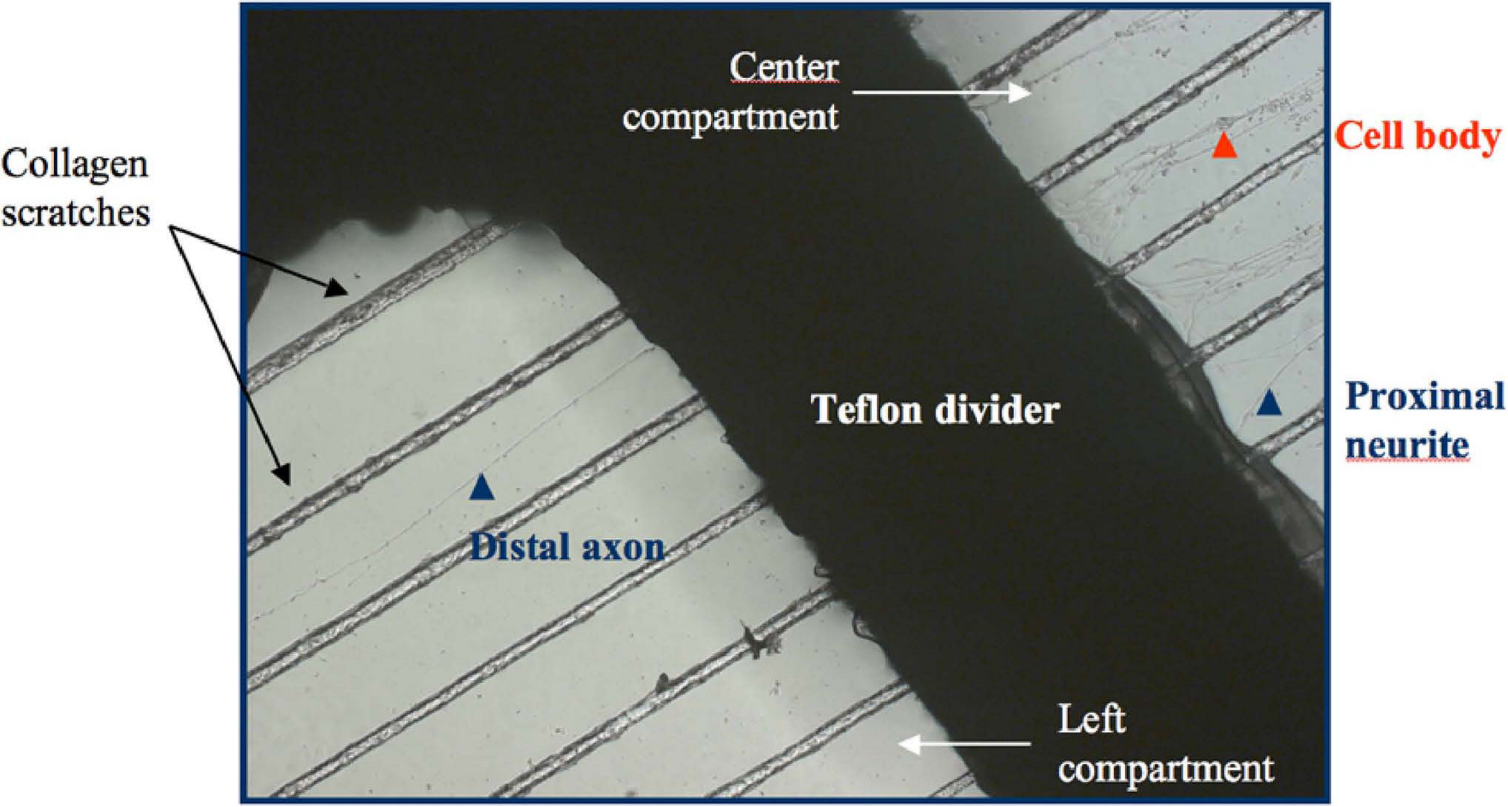
**Magnified detail of cultured sympathetic ganglion cells**. Ganglion cells growing in a compartmented chamber with an axon growing across the divider into the axon terminus chamber.

Cultured neurons grown in compartmented Campenot chambers [52] were used to demonstrate the direct relationship between ATF and the ability to reach the cell body via axonal transport (Figures 17 &18). FITC conjugated WGA was compared to Texas Red (Sulforhodamine 101 sulfonyl chloride) (TR) conjugated NGF in this model so that the fundamental efficacy and relative efficiency of derivatized ATFs for axonal transport and for promoting transport of the tripartite complex could be demonstrated definitively.

See Claude et al 1982 [53] for full method including sympathetic ganglion culture, Campenot chambers, assessment of receptor number and saturation. We used this experimental arrangement to compare transport of WGA-FITC to NGF-TR (Texas Red) to assess the impact of ATF (axonal transport facilitator) on transport. We then prepared fluorescent labeled WGA-dextran-FITC and NGF-dextran-TR for comparison in the Campenot chamber model.

##### Use of phage display iterative processing to generate synthetic ATFs [study 8]

By combining phage display technology with Campenot chamber technology, we were able to explore for and identify new purely synthetic ATFs. We harvested phage from the neuronal cell body after exposing the axons in sealed chambers to large numbers of phage variants. These phages were reexposed to second and third tier Campenot chamber sorting so that only well transported phage variants were selected and their surface variant proteins amplified and characterized by standard methods.

Phage display with an M13 phage library was carried out in a modification of the receptor panning method that has been described in detail previously [54-56]. We used inserted peptide sequences in a pIII library with the format CX_7_C denoting 7 amino acids in a disulfide constrained loop thereby offering 1.28 × 10^9 ^possible sequences. Three rounds of standard trkA receptor panning in open (uncompartmented) dishes were carried out. The selected output from the third round was placed in the distal axon terminus chamber in Campenot compartmented culture dishes. We allowed 15 minutes for incubation with phage at a concentration of 2.7 × 10^10 ^pfu/ml for adsorptive (receptor mediated) endocytosis, then washed the axon terminus chambers three times and then incubated for an additional 2 hours without phage to allow for axonal transport to the cell body chamber. Cell bodies were then recovered and phage was then isolated from the cell bodies by centrifugation, amplified and passaged again by reincubation with axon termini. After three rounds of transport, the collected phage peptide was sequenced, synthesized and labeled with FITC at the C-terminal end.

Once added to the side compartment it was taken up by the distal terminal neurites and axonally transported to the cell bodies. The peptide was coupled to the polymer vehicle and again demonstrated axonal transport to cell bodies that was blockable by colchicine as an axonal transport inhibitor as described above.

##### Use of transport inhibitors to confirm axonal transport basis of effect [study 9]

We sought to assess whether redistribution of the test agents was due to axonal transport rather than diffusion across the barriers, intra-membrane flow, or passive distribution and diffusion within the axoplasm after endocytosis. To test this we evaluated the effects of an axonal transport inhibitor on movement of WGA-dextran-FITC, NGF-dextran-TR, or 'Phage display generated ATF'-dextran (or polyethylene glycol)-FITC - tripartite molecules from the axonal compartment of Campenot chamber cultures to the cell body compartment. These studies were conducted with and without pre-treatment with colchicine (125 micromolar) - an inhibitor of axonal transport - for one hour prior to administering the tripartite [57,58]. The tripartite complex was incubated in the distal axon chamber of Campenot chamber cultures of sympathetic ganglia. We assessed FITC or TR fluorescence in the cell body chamber comparing cultures in which colchicine was included with the tripartite to those incubated without colchicine [53].

#### Effects of Intra-axonal Processing (project V)

##### Anti-gabapentin antibodies to demonstrate drug delivery [study 10]

To explore survival of gabapentin (1-aminoethyl-cyclohexane acetic acid - an analogue of GABA - gamma-aminobutyric acid) in rapid transport axonal vesicles, we injected the tripartite complex of WGA-dextran-gabapentin in rat biceps muscle and used gabapentin antibodies to confirm delivery of materials with preserved antigenicity into neuron cell bodies in the CNS after intramuscular injection in the periphery.

Antibodies to gabapentin were raised and used as a primary against sections of rat spinal cord and DRG excised and cryosectioned at 1, 4 and 7 days after administration of WGA-dextran-gabapentin and of dextran-gabapentin into biceps femoris muscle in rats. Secondary antibody with FITC was then evaluate by fluorescence microscopy.

##### WGA-HRP to demonstrate intact delivery of enzymes [study 11]

To assess survival of enzymes during transport in a primate model with axons up to 20 cm in length, we injected microgram quantities of wheat germ agglutinin (WGA) (*Triticum vulgaris*) conjugated to horseradish peroxidase (HRP) in microliter volumes in *Macaca fascicularis *epaxial (back) muscles (short transport distance) and hypaxial (abdominal) muscles (long transport distance). After three days, peroxidase enzymatic amplification of transported (HRP) in sectioned spinal cord with tetramethylbenzidine (TMB) staining of the reaction product [59,60] allowed evaluation by back-field fluorescent microscopy. The injections sites were also stained to accurately assess the distribution of remaining injectate in the source tissue.

Four adult *Macaca fascicularis *animals were anesthetized with 25 mg/kg ketamine and 2 mg/kg xylazine (IM) and in various combinations, erector spinae, transversospinalis, or rectus abdominis muscles were surgically exposed and injected with a 5% solution of lectin conjugated horseradish peroxidase (WGA-HRP, Sigma Chemical Co, St. Louis, MO). To sample a wide range of motor units while minimizing the possibility of tracer leakage to adjacent muscles, WGA-HRP was delivered via multiple, small intramuscular injections with either a 1 μl or 5 μl Hamilton syringe. A total of 0.5 to 2.5 μl was delivered to some various muscles (one to five injections of 0.5 μl each). During surgery, care was taken to preserve the integrity of the fascial membranes separating the various muscles as intact fascial membranes can form a barrier to the leakage of tracer from injected muscle [61].

Eighteen to seventy-two hours following WGA-HRP injection, the animals were reanesthetized with an overdose of pentobarbital sodium and perfused through the left ventricle with 1-2 liters of 0.9% saline, followed first by 1-2 liters of a fixative containing 0.5% paraformaldehyde, 2.0% glutaraldehyde, and 2.0% sucrose in a 0.1 M phosphate buffer and second by 0.5-1.0 liters of 10% sucrose in 0.1 M phosphate. The spinal cord was exposed, spinal nerves II-XXVI were identified and segments IX to XXIII were removed and stored, together with excised relevant muscles, for 1-3 days in a 20% sucrose solution of 0.1 M phosphate at 4°C.

The spinal cord was cut serially in 75 μm transverse sections on a freezing microtome. Frozen 75-100 μm sections of the muscles were also cut in transverse, sagittal, and coronal planes. Spinal cord and muscle sections were collected in a solution containing 30% sucrose and 30% ethylene glycol in 0.1 M phosphate buffer and stored for up to 1 week at 0°C.

Spinal cord and muscle tissue from all experiments was processed with tetramethyl benzidine (TMB) [59,62,63]. Every second or fourth section of brainstem tissue and every fourth or sixth section of muscle was reacted with TMB for 2-4 hours, mounted on gelatin-coated slides, air dried, and counterstained in neutral red. Spinal cord and muscle slides were examined in brightfield, darkfield, or polarized light microscopy to determine the extent of the injection site and resulting spinal cord label. Spinal cord and muscle sections with WGA-HRP label were photographed and traced.

##### Electron Microscopy to demonstrate WGA-dextran-magnetite in transport stream [study 12]

To confirm the location of WGA-dextran-magnetite in transport vesicles in axons, distributed by axonal transport, we carried out electron microscopy of rabbit sciatic nerve at appropriate time intervals for fast axonal transport after injection in gastrocnemius and anterior tibial muscles.

WGA-dextran-magnetite conjugate complex was prepared, purified, filter sterilized and injected. After allowing six hours to elapse, animals were sacrificed, perfused, and their upstream ipsilateral and untreated contralateral sciatic nerves were then excised and prepared for electron microscopy [31].

##### Autoradiographic assessment of [^59^Fe]-WGA-dex-mag location [study 13]

To additionally prove intraneural transport we made [^59^Fe]-dextran magnetite and carried out large format autoradiography using similar administration methods and timing as in the electron microscopy experiments.

This agent was produced as in study 2 above, including 250 microCuries of [^59^Fe] in the initial ferrite nanoparticle precipitation step. The injectate for each experiment represented a concentrate of about one tenth of the batch of [^59^Fe] particles so that 25 microCuries were delivered. The [^59^Fe]-WGA-dex-mag was administered in rabbits. As in the micro MRI imaging experiments, injection involved preparations concentrated to 15 mg Fe/ml injected into forearm musculature of rabbits using three sites, 50 microliters per site, using pre-puncture with an 18 gauge needle, introduction of a Hamilton syringe for injection, then superglue seal of the puncture site.

After a four day survival, the rabbits were sacrificed under deep barbiturate anesthesia by intracardiac perfusion with sucrose glutaraldehyde solution. The forelimb from the ipsilateral and contralateral side was then frozen, cut as an intact cross section, refrozen sealed with cellophane and applied to X-ray film in cassettes in a freezer at -4 degrees centigrade for 12 hours.

##### WGA-dex-mag MRI microscopy to demonstrate intact magnetite during transport [study 14]

Microscopic MRI (50 micron in plane resolution) was used to demonstrate the preservation of intact transport particles during axonal transport. WGA-dextran-magnetite was injected in rabbit gastrocnemius muscle with subsequent microscopic MRI. Significant hydrolysis of the magnetite superparamagnetic nanoparticles would have destroyed their relaxivity (contrast effect). Rabbits were used to provide an order of magnitude increase in distance transported relative to rats and to assure that the area of sciatic nerve imaged or examined was sufficiently distant from the injection site to reliably eliminate any local spread. Use of rabbits also extended the efficacy of microscopic MRI for evaluation of sciatic nerves *in vivo *due to the order of magnitude increase in body size and nerve size relative to rats while still accommodating the 12 centimeter inner bore size of the microscopic MRI scanner system. In the setting of MRI, physical limits on spatial resolution are presented by instrument system design factors (gradient coil strength, surface coil geometry) so increased animal size is the only effective compensation.

**Figure 19:**
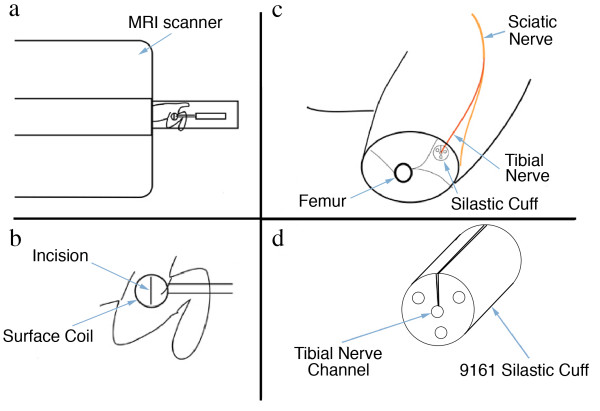
**Microscopic MRI silastic cuff imaging set up**. (**a**) The rabbit is positioned on gantry outside MRI scanner with surface coil over thigh. (**b**) Blow up shows the surface coil over the incision line. (**c**) The silastic cuff is shown in place on the tibial nerve in a cross section of the thigh. (**d**) The 9161 silastic cuff is shown with three outer channels for contrast gel standards and a center channel with access slit for the nerve to be imaged.

High field micro-MRI images were obtained with a 4.7 Tesla, 33-cm SISCO system fitted with a 100 miliTesla/meter high performance auxiliary gradient insert (12-cm inner bore). A single turn 2 cm surface coil was placed against the upper portion of the lower leg of 2 to 2.5 kg rabbits. The limb was taped to the side of the supporting cradle to minimize motion artifacts (Figure 19). The animals were maintained under balanced continuous intravenous infusion of an anesthetic mixture containing 1 mg of medazolam, 1.5 mg of fluanisone, and 50 μg fentanyl/ml at rates of 4 to 10 ml/h to achieve a deep anesthesia which further minimized motion from respiration.

The imaging sequence is a standard multi-slice spin-echo imaging sequence. To enable the determination of the apparent T_2_, multiple images of the same transverse slice were acquired with different TEs (echo times). The data were obtained in an interleaved manner; the variable parameter (TE) was arrayed for each phase-encoding step, thus averaging any effects due to motion within the duration of the experiment.

All images were acquired with a repetition time TR = 1.5 s over a 4 × 4 cm field of view (FOV) and a 2-mm slice thickness. Two hundred fifty-six phase-encoding steps were used and 512 data points were acquired giving a basic in plane image resolution of 156 by 78 μm. For image analysis the data were zero filled to 1024 by 1024 and Fourier transformed after applying a 2D Gaussian filter that attenuated the high-frequency time-domain components by 6 dB. This clarified the delineation of the very small regions of interest (ROIs) (typically 0.001 to 0.003 cm^2 ^for nerves) but did not significantly change the average pixel intensities measured.

To establish persistence of the ferrite during transport we implanted in rabbits a four channel silastic chamber (cast from 9161 elastomer - Dow Corning) (1 cm diameter, 2 cm in length with channels left by casting the chamber around the tips of four micropipettes) in which three chambers were filled with polyacrylamide gels containing various concentrations of ferrites and fourth (with an external longitudinal slit) was placed around the nerve (see Figures 8, 19). The silastic is completely free of MRI signal providing a dense black background for the imaged channel contents. The chamber was secured in a subcutaneous location below a 2 cm diameter surface coil which afforded 100 micron spatial resolution. Spin echo imaging with the various TE at various echo times was employed to measure T2 in the nerve chamber and compare to the surrounding standards in a series of images and measurements as time after intramuscular injection progressed. Comparisons were made between the steady T2 value of nerve in uninjected animals vs animals with prior intramuscular injection (gastrocnemius) with the WGA-dex-Fe or WGA-dex agents at various times from one to eight hours after injection. Rabbits were implanted, injected and imaged under continuing intravenous general anesthesia and then sacrificed immediately following the imaging studies.

Injection involved preparations concentrated to 5-15 mg Fe/ml injected into calf and anterior tibial musculature using three to five sites, 50 microliters per site, using pre-puncture with an 18 gauge needle, introduction of a Hamilton syringe for injection, then superglue seal of the puncture site.

##### High resolution MRI of transport for contrast effect & particle preservation [study 15]

To further confirm the axonal transport nerve contrast effect at appropriate time frames, we used serial solenoid coil imaging of the upper arm of a rabbit after injection of WGA-dextran-magnetite with no surgical alterations. Instead we relied on various manipulations of pulse sequence and on magnet parameters to accomplish nerve identification and to allow a bright T2 signal from nerve [44,45,64]. Then, by injecting the relevant musculature, we could observe a gradual decrease in the physiologically expected MRI nerve image signal.

The basic method is similar to study 14 except that the system was used for high resolution MRI of rabbits who had the WGA-dex-mag injections rather than micro MRI. For this purpose, we used a solenoid coil to image the forearm of essentially as described in Howe et al [44].

Injection involved preparations concentrated to 5-15 mg Fe/ml injected into forearm flexor musculature in rabbits using three to five sites, 50 microliters per site, using pre-puncture with an 18 gauge needle, introduction of a Hamilton syringe for injection, then superglue seal of the puncture site.

Images were obtained with a 4.7 Tesla, 33-cm SISCO system fitted with a 100 miliTesla/meter high performance auxiliary gradient insert (12-cm inner bore). A solenoid coil was placed around the proximal portion of the forelimb of 2 to 2.5 kg rabbits. The limb was taped to the side of the supporting cradle to minimize motion artifacts. The animals were maintained under balanced continuous intravenous infusion of an anesthetic mixture containing 1 mg of medazolam, 1.5 mg of fluanisone, and 50 μg fentanyl/ml at rates of 4 to 10 ml/h to achieve a deep anesthesia which further minimized motion from respiration.

The imaging sequences included either a standard multi-slice spin-echo imaging sequence or a multi-slice STIR (short tau inversion recovery) sequence.

### Targeting and Pharmacological Efficacy (aspect C)

#### Clinical Target Access (project VI)

To clarify the capability for selective targeting of neuronal sub-populations, we conducted a series of evaluations in rats with three types of fluorescent labeled compounds.

For assessment of extent of access to dorsal root ganglion (DRG) cells and to dorsal root entry zone of Lissauer regions I and II from the periphery, we injected FITC labeled WGA in biceps femoris muscle and Tetramethylrhodamine isothiocyanate (TRITC) labeled WGA in foot pad. On each of days 1-28 following injection, dorsal root ganglia were collected, sectioned and analyzed with fluorescence microscopy for counts of total cells and of cells with transported FITC.

##### Ventral horn/Intermediolateral columns [study 16]

Access to the motor anterior horn cells and to autonomic intermediolateral column cells was investigated similarly by histological evaluation of spinal cord sections after WGA-FITC intramuscular administration in rats.

Intramuscular injection of WGA-dextran-FITC was followed by sacrifice of the animals at 8 to 72 hours after injection. Animals were sacrificed and perfused, spinal cord was excised, roots were counted, and appropriate levels were then sectioned, mounted and stained for histological analysis by fluorescence microscopy. This followed the methodology of older studies [65], but used the larger tripartite complex being evaluated for drug delivery to assure that it would reach the expected and desired targets.

##### Dorsal Root Entry Zone [study 17]

To assess access to the dorsal root entry zone Lissauer regions I and II from the periphery, we injected Tetramethylrhodamine isothiocyanate (TRITC) labeled WGA in biceps femoris muscle, gastrocnemius, anterior tibialis and in foot pad. Spinal cord was sectioned and analyzed with fluorescence microscopy for TRITC.

The duration of time to sacrifice after injection was extended to 24 - 72 hours and the ipsilateral dorsal root entry zone was assessed for evidence of filling of the proximal axons of dorsal root ganglion cells.

##### Foot pad/biceps to Dorsal Root Ganglion [study 18]

For assessment of extent of access to dorsal root ganglion (DRG) cells and to dorsal root entry zone of Lissauer regions I and II from the periphery, we injected FITC labeled WGA in biceps femoris muscle and Tetramethylrhodamine isothiocyanate (TRITC) labeled WGA in foot pad. On each of days 1-28 following injection, dorsal root ganglia were collected, sectioned and analyzed with fluorescence microscopy for counts of total cells and of cells with transported FITC. This allowed for assessment of the degree to which different cell populations could be accessed from the two different types of injection site.

##### Peripherin labeling for verification of access to nociceptor neurons [study 19]

In order to demonstrate that the cells in the dorsal root ganglia reached were nociceptors, we used post-staining with peripherin antibodies [66] to identify nociceptor neurons to look for colocalization with neurons labeled by tripartite WGA-dex-FITC injected in the periphery.

#### Distinctive Pattern of Distribution (project VII)

##### Whole body distribution - [^125^I]-WGA, [^125^I]-NGF, [^59^Fe]-WGA-dex-mag [study 20]

To explore the whole body and tissue distribution of axonally targeted agents and to identify the impact of tripartite design factors on the distribution, we injected [^125^I] labeled wheat germ agglutinin (WGA), [^125^I] labeled nerve growth factor (NGF), or dextran coated 5-15 nanometer [^59^Fe]-magnetite (ferrite) particles [46,67] conjugated to WGA (WGA-dex-mag) in hindlimb and forelimb musculature of rats with varying doses and measured concentrations in seventy tissues after survival times varying from one to five days.

*Identification of experimental factors affecting trans-neuronal distribution (protocol 20a)*

[^125^I]-WGA (wheat germ agglutinin) was obtained from ICN (specific activity = 25 mCi/mg) and concentrated with Centricon-10 (Amicon) centrifugal ultrafilters to a concentration of 333 μg/ml (8.9 μM) with a specific activity of 25 μCi/μgm in 0.1 M phosphate buffer, pH 7.4 (~7 μCi/μl). Twenty three rats (200-300 gm) were given water with Lugol's iodine (0.05% KI in water) for at least 3 days prior to the injection and were maintained on this water, one animal per cage, during a survival period of two to ten days in grid floor cages with twice daily changes of litter. Some animals were maintained in Nalgene metabolic cages in order to prevent ingestion of [^125^I] contaminated urine and feces and to permit daily collection of urine and feces for assay.

Concentrated [^125^I]-WGA injected percutaneously into rat forearm or hindlimb muscle in various microliter quantities. Introduction of the injectate was done in several different ways: 1) by incision access to muscle with suture closure, 2) by incision and using methacrylate to seal the incision site, 3) by skin puncture with an 18 gauge needle followed by Hamilton needle introduction then methacrylate closure of the puncture site, 4) percutaneous by needle puncture followed by methacrylate closure. The puncture sites (in 2, 3, & 4) were sealed with methacrylate superglue immediately upon removal of the needle. This measure was taken to limit oral uptake by licking of the wound.

Intramuscular targets were varied to include forearm (15 animals), calf (2 animals), or calf and anterior compartment of distal lower extremity (7 animals) or in posterior thigh muscle mass (4 animals) to assess sites which might offer either greatest muscle volume (thigh) or highest innervation ratio of axons and muscle spindles per unit of muscle (forearm) To compare with other models for tracer administration, distribution was assessed after handpad skin, tongue, vibrissal and cerebral cortex injections.

For the intramuscular injections, Hamilton syringes partially loaded with paraffin oil were used to obtain precise injection quantity. Under general anesthesia induced with intraperitoneal barbiturate, the site of injection was shaved, the skin was either opened over 3-4 mm with a scalpel for direct visualization of the target muscle or first punctured with a beveled 18 gauge needle. A 26S gauge Hamilton needle was introduced into the selected muscle mass. Injection sites were either in the forearm or calf. Injections were done slowly, delivering volumes of 0.1-0.5 μl in a series of locations in a selected muscle (without removing the needle from the muscle) over five minutes to minimize back diffusion. The largest total volume of injection was 6 μl for forearm sites and 10 μl for hindlimb sites.

In animals used in nerve intervention studies, the nerve was identified surgically under deep barbiturate anesthesia using an operating microscope. The nerve was either firmly crushed in forceps or crushed and then suture ligated with the lesion placed at a point at least two centimeters proximal to the muscle to be injected. After assuring hemostasis, the wound was then sewn closed and sealed over with methacrylate glue and the tracer injection then carried out via a separate puncture site which was also sealed with methacrylate glue.

The duration of time for distribution of the injected agent was varied from three to ten days after which animals were sacrificed with intraperitoneal barbiturate overdose, and, after withdrawing a blood sample by intracardiac puncture, perfused with phosphate buffered saline (PBS) to clear virtually all of the blood from the vasculature, and then with cold PBS paraformaldehyde solution. After perfusion, the animals were dissected and 70 different tissue specimens including lymph nodes, liver, lung, muscle, ipsilateral and contralateral peripheral nerve, spinal root/dorsal ganglia, and various spinal cord segments were collected.

The various tissues were sealed immediately in microfuge tubes for gamma counting with a Beckman Bio-Gamma counter within 48 hours of the time of perfusion. After counting, the samples, which remained in sealed microfuge tubes, were weighed with a Sartorius microgram balance. For specimens above 10 milligrams, an average standard weight for the microfuge tube was subtracted from the total weight, while for specimens below 10 milligrams, the specimen was removed from the tube for weighing.

The biodistribution trials were varied (as noted above) to assess optimal technique and to explore the effect of dosage and survival time (Figure 15). The variables explored include the dose, site, and technique of injection, as well as survival time, and animal maintenance conditions. For each animal, the raw counts per minute from each tissue sample was divided by the weight of that sample, thus yielding figures of cpm/mg for each tissue. These raw distributions can be compared from animal to animal, but to emphasize differences in the pattern of distribution rather than differences in pure magnitude, the results for each animal were standardized for comparison by dividing all results for a given animal by the cpm/mg in blood for that animal. This results in a dimensionless figure proportional to concentration in tissue relative to concentration in blood for each tissue in each animal.

Data are arrayed in chronological experimental sequence order. Experiments (***a***) and (***b***) used incision with suture closure and 3 day survival - these had relatively high distribution to liver and kidney apparently related to leakage and licking of the wound site, ***c ***is similar but used a longer survival time - this resulted in higher DRG accumulation, and lower liver and kidney levels apparently due to progressive metabolism after the wound had healed with no ongoing ingestion. In (***d***), a superglue seal was used on the incision and animals were kept in a metabolic cage to minimize ingestion of any excreted tracer - this resulted in a relatively higher level of nerve and DRG tracer, and very low distribution in liver, kidney, or stomach. In (***e***), (***f***), and (***g***), the [^125^I]-WGA was concentrated into one tenth the volume and this also decreased the liver, kidney and stomach levels apparently because the smaller volumes decreased leakage through the healing wound - this also resulted in much higher levels of delivery to nerve and DRG apparently because of a decrease in leakage of the injectate from the muscle into sub-cutaneous non-innervated areas as well ((***g***) was performed with a low dose and the results were multiplied by 50 for purposes of visualization on this graph). Hindquarter injections ((***h***) to (***m***)) were more convenient for multiple injections because of the larger available muscle mass compared to forelimb in these animals. In (***h***), a large dose with 20 injection locations of 0.5 microliters each, longer survival led to higher DRG concentration relative to nerve. In (***i***) and (***j***), ten small injections were done resulting in greater widespread distribution in the muscle as well as good retention in the muscle resulting in the highest nerve and DRG distributions obtained. In (***k***), a 10 gm-cm spinal cord injury was applied and in (***l***), a 25 gm-cm spinal cord injury was applied - the total amount transported was decreased at 10 gm-cm so that the amounts are multiplied by 5 to yield visible graph data even though the typical transport distribution was still seen, however at a 25 gm-cm injury, little or no transport occurred (liver, kidney and stomach distribution appear increased because of the multiplication for visibility purposes). In (***m***), the very long survival of 10 days shows evidence of eventual metabolism out of the nerve and DRG (multiplied by 10 to emphasize the differential distribution despite overall very low retention).

*[^125^I]-NGF studies to evaluate effect of ATF on distribution (protocol 20b)*

Similar methodology was used for [^125^I]-NGF (nerve growth factor) using 4 day survival, hindlimb injections, three 1.0 microliter injections delivered by percutaneous injection and superglue seal in rats.

*[^59^Fe]-WGA-dex-magnetite to assess effects of size on distribution (protocol 20c)*

The [^59^Fe]-WGA-dex-mag was administered in rabbits. As in the MRI imaging experiments, injection involved preparations concentrated to 5-15 mg Fe/ml injected into calf and anterior tibial musculature using three to five sites, 50 microliters per site, using pre-puncture with an 18 gauge needle, introduction of a Hamilton syringe for injection, then superglue seal of the puncture site. This agent was produced as in study 2 above, but including 250 microCuries of [^59^Fe] in the initial ferrite nanoparticle precipitation step. The injectate for each experiment represented a concentrate of about one tenth of the batch of [^59^Fe] particles so that 25 microCuries were delivered.

##### Gamma camera imaging of [^131^I]-WGA to evaluate local injection effects [study 21]

To compare amounts transported versus amounts remaining at the site of injection for purpose of radiolabel imaging, we injected [^131^I]-WGA in the gastrocnemius and anterior tibial compartment of rabbits, then imaged at intervals with a gamma camera.

WGA was periodate labeled with [^131^Iodine], washed and concentrated in centrifugal ultrafilters, then injected in the forearm muscles of rabbits. Injection were by Hamilton syringe involving an 8 microCurie injection as two 1.0 microliter injections, a 24 microCurie injection as three injections of 2 microliters and a 60 microCurie injection as five injections of 3.0 microliters. The injection sites were sealed with superglue. After the elapse of four days, the animals were placed deep barbiturate anesthesia and then imaged in a clinical gamma camera.

##### [^14^C]-Gabapentin for evaluation of distribution of axonally transported gabapentin [study 22]

To document the efficacy of the carrier for delivery of a small molecule pharmaceutical *in vivo*, we prepared [^14^C]-labeled gabapentin and conjugated it to polymer without ATF and to polymer conjugated to ATF, administered these by intramuscular injection, then counted various ipsilateral and contralateral muscle and nerve tissues.

The use of radiolabeled [^3^H]-gabapentin has played an important role in studies of gabapentin localization [68,69]. We used [^14^C]-gabapentin [70] for distribution studies essentially as in study 18 above comparing [^14^C]-gabapentin-dextran to WGA-dextran- [^14^C]-gabapentin to assess the impact of the ATF on resulting gabapentin distribution. We chose to use [^14^C]-gabapentin rather than [^3^H]-gabapentin because of the expectation of less exchange of the labeled carbon relative to exchange of a labeled hydrogen with other molecules and also to facilitate gamma counting in dissected tissues due to the greater energy of the gamma emission.

##### Paw withdrawal latency for clinical efficacy evaluation in reduction of hyperalgesia [study 23]

To evaluate potential clinical efficacy for analgesia *in vivo*, an affinity purified agent comprising WGA-dextran-gabapentin was used in a well characterized hyperalgesia model [71]. In each of three repetitions of the experiment, groups of six rats had one of four treatments: injection of into the muscle and paw of the affected hindlimb of (1) - the full tripartite agent (WGA-dextran-drug), (2) - dextran-drug with no axonal transport facilitator or (3) - an equivalent dose of free drug or (4) - tripartite agent injected subcutaneously in the cervical fat pad for systemic delivery. Total doses of gabapentin were 75 micrograms in adult 200 gm Sprague-Dawley rats. Paw withdrawal latency in the ipsilateral and unaffected contralateral limb was measured (a total of 72 animals).

The molecular complex (WGA-dextran-gabapentin) was configured to deliver the anticonvulsant, gabapentin, to selected dorsal root ganglia by retrograde axonal transport after injection into peripheral tissue innervated by the target nerves. The efficacy of this agent was evaluated *in vivo *using the chronic constriction injury model of neuropathic pain. Paw withdrawal latency (PWL) to thermal stimuli was measured in the ipsilateral and contralateral limbs. Injection of the ATF drug complex (total gabapentin dose; 75 mg/200 gm rat) or relevant control was into hindlimb muscle and paw with superglue seal of injection site. Animals were evaluated for PWL prior to surgery, on the date of surgery, and at two day intervals for twenty five days.

All animal work was carried out under strict compliance with the relevant Institutional Review Board or Home Office License requirements at the various institutions involved in the work.

## Authors' contributions

AGF - Overall planning, writing, experimental design for all aspects, carried out many of the experiments along with various co-authors for tracer work, chemical synthesis, microMRI, pharmacological distribution work. GTW - Planning and completion of *in vivo *paw withdrawal latency work, histochemistry, targeted labeling studies. MB - Initial development of biochemical methods for conjugation, histology and histochemical work, oversight and implementation of assay, histology, and chemical synthesis. MF - Advanced development of methods of conjugation, participation in synthesis of compounds. FAH - helped design and participated in micro-MRI experiments. MDR - developed basic intramuscular injection model and participated in histochemical studies. AJS - developed analysis methods for intramuscular injection tracer/histology. TWD - co-developed and participated in initial complex tracer conjugation parameter studies. CA - participated in chemical strategies and syntheses for various pharmaceutical conjugation. RM - designed and participated in work for *in vivo *paw withdrawal latency work. JRG - participated in biochemistry for magnetite chemistry and micro-MRI work. BAB - supervision and design participation for overall project and writing. AMLL - design and experimental participation for viral assay work, chemical synthesis work, overall project management participation. All authors read and approved the final manuscript.

## Authors' Information

**AGF **MD, PhD, FRCS - Medical Director, Institute for Nerve Medicine, Santa Monica, CA; Inventor US patent 6,562,318; **GW **DPhil - Associate Director Wyeth Research; **MB **DPhil - Director of Research, Spinal Research, Guildford, Surrey, UK; **MF **PhD - Research Chemist, Astex Therapeutics; **FAH **DPhil - Senior Lecturer in MRI, St. George's University of London; **MDR **PhD - Research Scientist, Carmell Therapeutics, Pittsburgh, PA, USA; **AJS **PhD - Assistant Professor, Department of Physiology, Emory University; **TWD **PhD - Professor of Anthropology, University of California at Berkeley; **CA **DPhil - Professor of Chemistry, University of Cambridge; **RM **MD - Consultant in Chronic Pain/Anesthesiology; **JRG **DPhil - Editor-in-Chief - *NMR in Biomedicine*; **BAB **MD - Professor of Neurosurgery, St. George's University of London; **AMLL **MD - Professor of Medicine, University of Cambridge, Associate Editor- Retrovirology.
